# BnaA07.SUC2 regulated by BnaA05.MYC2 in jasmonate pathway promotes oilseed rape susceptibility to *Plasmodiophora brassicae*

**DOI:** 10.1371/journal.ppat.1014199

**Published:** 2026-05-05

**Authors:** Jiaqi Liu, Ziyue Xu, Yanlin Li, Shifan Wu, Zihan Ni, Xinmeng Li, Peiyao Li, Xiaoqun Liu, Xinyi Dang, Tianyu Wang, Jingyan Fu, Maolin Wang, Rui Wang

**Affiliations:** Key Laboratory of Bio-Resource and Eco-Environment of Ministry of Education, College of Life Sciences, Sichuan University, Chengdu, China; Centre National de la Recherche Scientifique, FRANCE

## Abstract

Biotrophic pathogens depend on host carbon sources for proliferation. Here, we identified *BnaA07.SUC2* in oilseed rape (*Brassica napus* cv. Westar), which encodes a plasma membrane-localized proton-dependent sucrose transporter. Its expression is markedly induced in roots during the late stages of *Plasmodiophora brassicae* infection. The *Arabidopsis thaliana suc2* mutant exhibited a strong clubroot-resistant phenotype, and functional complementation with *BnaA07.SUC2* restored susceptibility to *P. brassicae*. Overexpression of *BnaA07.SUC2* in oilseed rape significantly increased sucrose accumulation and disease susceptibility, whereas CRISPR/Cas9-mediated knockout of *BnaA07.SUC2* enhanced clubroot resistance. Furthermore, yeast one-hybrid, dual-luciferase, and electrophoretic mobility shift assays showed that BnaA05.MYC2 directly binds to the *BnaA07.SUC2* promoter and represses its expression. Overexpression of *BnaA05.MYC2* in oilseed rape enhanced clubroot resistance, which was accompanied by reduced *BnaA07.SUC2* transcript levels. Conversely, co-overexpression of *BnaA07.SUC2* in the *BnaA05.MYC2*-overexpressing oilseed rape background restored susceptibility, indicating that *BnaA05.MYC2* promotes clubroot resistance by repressing *BnaA07.SUC2*. *BnaA05.MYC2* expression is induced by jasmonate (JA) signaling. JA signaling is activated during early infection but suppressed in later stages, and accordingly, *BnaA05.MYC2* expression is upregulated initially but downregulated later. This downregulation relieves the repression of *BnaA07.SUC2*, thereby enhancing sucrose supply to the pathogen. Our study identifies the SUC transporter BnaA07.SUC2 and its regulator BnaA05.MYC2 as key susceptibility components in *P. brassicae* pathogenesis, providing important insights into the interaction between *P. brassicae* and its host plant.

## Author summary

*Plasmodiophora brassicae* is an obligate biotrophic pathogen that causes severe yield losses in cruciferous plants. Its life cycle depends entirely on the acquisition of energy from its host. The present study found that a sucrose transporter gene, *BnaA07.SUC2*, in oilseed rape significantly responded to infection by *P. brassicae*. Further analysis showed that *BnaA07.SUC2* encodes a proton gradient-dependent sucrose uptake transporter localized to the plasma membrane. Overexpressing *BnaA07.SUC2* in oilseed rape significantly elevate its root sucrose content and susceptibility to *P. brassicae*. Subsequent studies revealed that *BnaA07.SUC2* expression is suppressed by the transcription factor BnaA05.MYC2, whose expression is positively regulated by the jasmonate (JA) signaling pathway. During infection, the JA pathway in oilseed rape roots is activated before 14 dpi and suppressed after 14 dpi. At the later stage, the suppressed JA pathway results in a significant reduction in *BnaA05.MYC2* expression thereby leads to derepression of *BnaA07.SUC2*, supplying abundant sugars to the pathogen and facilitating disease development.

## Introduction

Sucrose produced in source leaves is loaded into the phloem and transported to sink tissues, where it is unloaded to support plant growth and development. Sugar transporters play a key role in this process of sucrose loading, transport, and unloading [1–6]. In contrast, following infection, plant pathogens must divert host carbon resources such as sucrose and its cleavage products to support their proliferation and disease progression [7]. Notably, obligate biotrophs, through long-term co-evolution, have developed sophisticated strategies to simultaneously evade host immune recognition and efficiently acquire photoassimilates [8,9]. A key mechanism involves hijacking host sugar transporters to enhance nutrient flux toward infection sites, thereby facilitating their own colonization [10,11].

Previous research has shown that obligate biotrophic pathogens often target and manipulate host sugar transporters, particularly those of the Sugars Will Eventually Be Exported Transporters (SWEET) and Sugar Transport Protein (STP) families [12]. TaSWEET14d, a member of the wheat SWEET family localized to secretory vesicle membranes, mediates sugar transport during *Puccinia striiformis* f. sp. *tritici* (*Pst*) infection. Following pathogen invasion, TaSWEET14d transports cytosolic sugars into secretory vesicles; these vesicles subsequently fuse with the extrahaustorial membrane (EHM), releasing sugars into the fungal extrahaustorial matrix and thereby providing direct nutrients for the pathogen. Additionally, TaSWEET14d may also reside in the EHM, where it could facilitate sugar transport from the host cytoplasm to the extrahaustorial matrix, ensuring an adequate carbon supply for fungal growth [13]. In the wheat-stripe rust interaction, STP members, which mediate extracellular sugar retrieval and cellular uptake, are manipulated by *Pst* to facilitate the pathogen’s proliferation. Loss of *STP13* function in the wheat variant *Lr67*^*res*^ results in failure of sugar retrieval, thereby conferring broad-spectrum resistance against biotrophic pathogens [14]. Similarly, overexpression of the plasma membrane-localized wheat hexose/sucrose transporter *TaSTP3* enhances wheat susceptibility to stripe rust [15]. *TaSTP6* expression is upregulated by abscisic acid, which in turn facilitates *Pst* infection [16]. Furthermore, STPs also contribute to powdery mildew susceptibility in *Arabidopsis thaliana*. Upon infection, the sugar transporter AtSTP8 is recruited to the EHM, where it facilitates sugar acquisition by fungal haustoria. Overexpression of *AtSTP8* increases hexose accumulation in leaves and enhances susceptibility to powdery mildew [17].

The Sucrose Transporter (SUC/SUT) family plays critical roles in sucrose loading, long-distance transport, and consequently in plant growth, development, and fruit ripening [18–20]. Beyond these classical physiological functions, they also serve as important regulators mediating plant-microbe interactions. For instance, the maize sucrose transporter ZmSUT1 competes directly with the transporter UmSRT1 from *Ustilago maydis* for host sucrose. Efficient uptake by ZmSUT1 significantly limits pathogen nutrient acquisition, thereby conferring host resistance [21]. Similarly, in tomato, silencing of *SlSUT2* limits the host’s capacity to recapture sucrose, resulting in enhanced carbon allocation to arbuscular mycorrhizal fungi and consequently promoting their proliferation [22]. Collectively, these studies suggest that SUC/SUTs, similar to STPs, act as potential facilitators of obligate biotrophic pathogens by enhancing sugar uptake for these parasites’ benefit. Nonetheless, the precise molecular mechanisms by which SUTs modulate host sugar flux to support pathogen development remain unclear.

Sugar signaling and transport are influenced by interactions with plant hormones, such as jasmonic acid (JA). In tomato, low red:far-red light (R:FR) or *phyB* mutation suppresses JA signaling, causing soluble sugar accumulation in leaves. This creates a sugar-rich environment that favors the growth of *Botrytis cinerea*, thereby promoting disease development. Consistently, the JA biosynthesis mutant *def1* in tomato exhibits elevated soluble sugar levels, and this phenotype is rescued by exogenous methyl jasmonate (MeJA) application [23]. These results indicate that JA signaling negatively regulates sugar accumulation in plants. MYC2 is the core transcription factor mediating JA-responsive gene expression. In the absence of jasmonoyl-isoleucine (JA-Ile), JAZ proteins repress MYC2 activity; upon JA-Ile accumulation, the SCF^COI1^ complex mediates JAZ degradation, releasing MYC2 and activating downstream gene expression [24–27]. In plant growth and development, OsMYC2 regulates rice flowering time via the sugar transporter *OsSWEET4* [28]. Nevertheless, whether JA-modulated sugar metabolism in plant-pathogen interactions is achieved through MYC2-dependent regulation of sugar transporters remains largely unknown.

*Plasmodiophora brassicae* is a plasmodiophorid protist belonging to the class Phytomyxea within the eukaryotic supergroup Rhizaria. As a soil-borne, obligate biotrophic parasite, it proliferates within host cells as a multinucleate plasmodium [29,30]. This pathogen causes clubroot disease, which impairs water and nutrient uptake by cruciferous plants, leading to severe global yield losses [31,32]. In *A. thaliana*, the *sweet11 sweet12* double mutant exhibited significantly reduced pathogen colonization and disease severity. Complementation of the *sweet11/12* mutant with either *SWEET11* or *SWEET12* restored both pathogen development and gall size to levels indistinguishable from the wild type [33]. These findings demonstrate that *P. brassicae* development depends on host sugar transporters. However, whether JA signaling mediates the competition of *P. brassicae* for host sugars via sugar transporters remains unclear.

This study conducted a comprehensive investigation into the role of SUC/SUT in *P. brassicae* pathogenesis. Transcriptome analysis showed that *BnaA07.SUC2* was significantly upregulated during the later stages of infection in oilseed rape. This upregulation corresponded to the pathogen’s peak proliferation phase, which required substantial energy from the host. Functional validation demonstrated that overexpression of *BnaA07.SUC2* consistently enhanced pathogen colonization and host susceptibility to *P. brassicae*. Further mechanistic investigation revealed *BnaA05.MYC2*, a JA-responsive transcription factor, as a direct negative regulator of *BnaA07.SUC2*. During later infection stages, the decline in host JA levels reduced *BnaA05.MYC2* expression. This downregulation relieved the repression of *BnaA07.SUC2* expression, thereby enhancing sucrose import into the cytoplasm to supply carbon and energy for pathogen proliferation.

## Result

### *P. brassicae* invasion alters assimilate transport routes in oilseed rape roots

To investigate the impact of *P. brassicae* infection on assimilate transport routes in oilseed rape roots, the fluorescent symplastic tracer 5(6)-carboxyfluorescein diacetate (CFDA) was loaded into the outer epidermal cells at different time points post-infection. The distribution of fluorescence was analyzed to examine pathogen-induced alterations in these transport routes [34].

**Fig 1:**
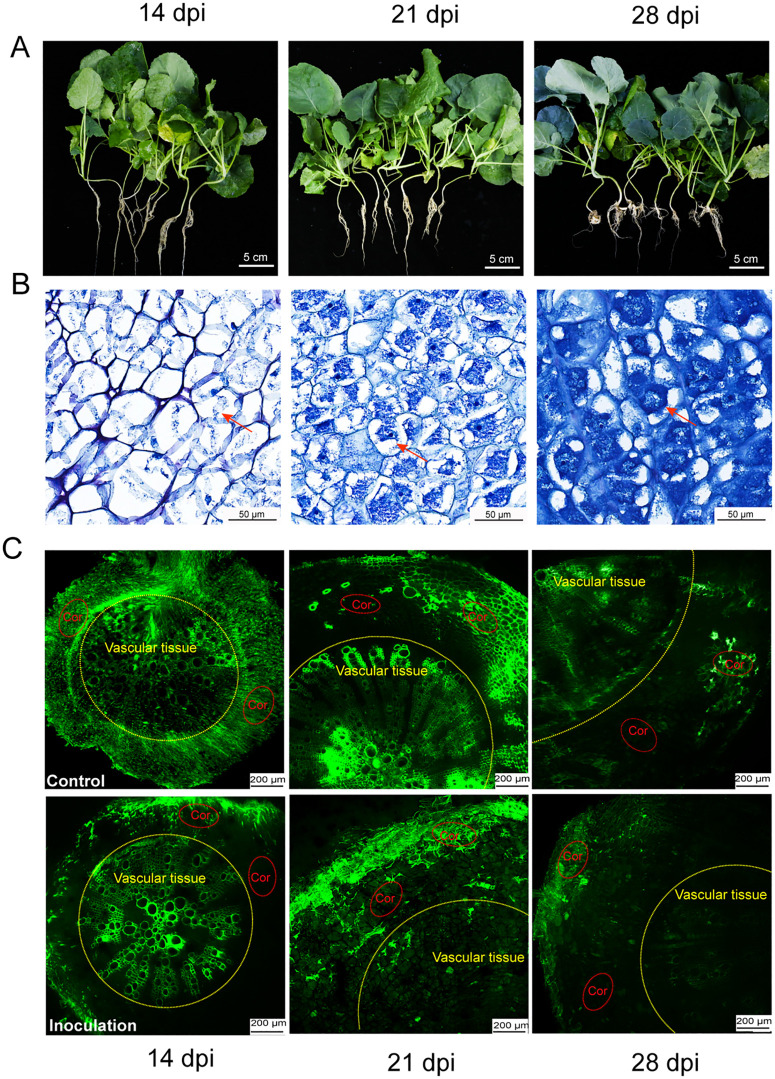
Symptom development and changes in photoassimilate transport routes in oilseed rape roots during *P. brassicae* infection. **(A)** Clubroot phenotypes of the susceptible oilseed rape cultivar ‘Westar’ at different days post-inoculation (dpi). Plants were inoculated with field-collected resting spores of *P. brassicae*. Scale bar = 5 cm. **(B)** Cross sections of clubroots corresponding to the tissues in **(A)**, stained with toluidine blue. Red arrows indicate plasmodia of *P. brassicae*. Scale bar = 50 μm. **(C)** Changes in assimilate transport routes in the mature zone of the primary root of ‘Westar’ at different infection stages, visualized by CFDA tracer loading. Scale bars = 200 μm. Yellow dashed circles indicate the vascular tissue region, while red dashed circles indicate the cortical region. Regions with fluorescence represent symplastic transport of assimilates, whereas regions without fluorescence represent apoplastic transport.

At 14 days post-inoculation (dpi), infected roots exhibited no obvious morphological changes (Fig 1A). However, microscopic examination of toluidine blue-stained root cross-sections revealed that *P. brassicae* had colonized the cortical cells but had not yet initiated active proliferation (Fig 1B and S1 Fig). Fluorescence signal was detected in both the cortical cells and the vascular tissues of infected and control roots (Fig 1C), indicating that the symplastic transport route predominated at this stage. By 21 dpi, morphological changes were observed in host roots, with the primary root exhibiting slight swelling (Fig 1A). Microscopic examination of root cross-sections revealed that *P. brassicae* had significantly proliferated compared with that at 14 dpi, with the colonization area expanding within infected cells (Fig 1B and S1 Fig). Fluorescence tracing revealed that in control roots, fluorescent signals remained diffusely distributed within the vascular tissues, indicating sustained symplastic transport. In infected roots, however, fluorescence signals were largely confined to cortical cells, with only weak signal remaining in vascular tissues (Fig 1C). This suggests that *P. brassicae* infection induces a transition from predominantly symplastic to apoplastic transport at this stage. At 28 dpi, infected roots exhibited pronounced morphological alterations, characterized by tissue deformation and the formation of typical clubroots (Fig 1A). Microscopic observation revealed that *P. brassicae* colonization within root cells had further increased, gradually occupying the entire cell and approaching mature resting spore formation (Fig 1B and S1 Fig). Fluorescence signal detection showed that in control roots, fluorescent signals were weakly detectable in both cortical and vascular tissues, suggesting a combined symplastic and apoplastic transport route at this stage. In infected root tissues, however, fluorescence signals were only weakly detectable in cortical cells, indicating that symplastic transport is largely impaired and apoplastic transport predominated at this stage (Fig 1C).

Together, these results suggest that a significant shift from symplastic to apoplastic transport occurs in infected host roots starting at 21 dpi, indicating that sucrose transporters play a central role in regulating sugar partitioning during this process.

### *BnaA07.SUC2* is proposed as a key target in *P. brassicae* sugar acquisition from the host

**Fig 2:**
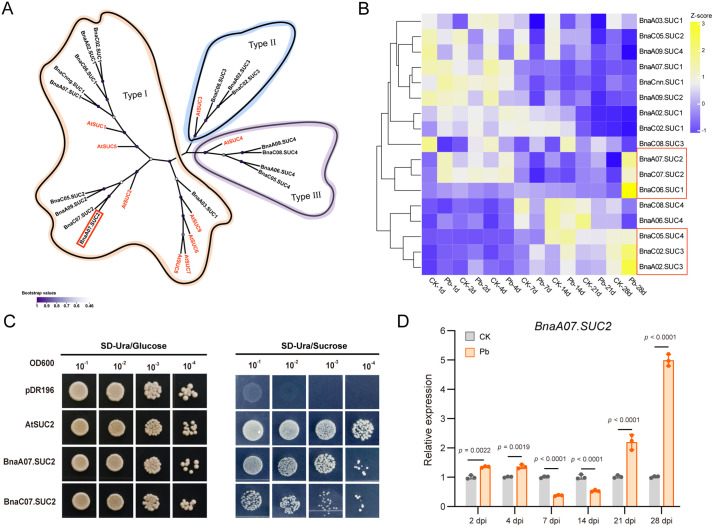
*BnaA07.SUC2* is involved in sugar acquisition by *P. brassicae* from the host. **(A)** Phylogenetic analysis of 17 differentially expressed oilseed rape *SUC* genes derived from transcriptome sequencing of *P. brassicae*-inoculated roots, with 9 *SUC* genes from *Arabidopsis thaliana* included as references. **(B)** Heatmap showing expression profiles of the 17 *SUC* genes in *P. brassicae*-infected (Pb) and control (CK) roots. Genes in red boxes were selected as candidates based on their significantly induced expression at 21 and 28 dpi. Data are shown as transcripts per million (TPM) values obtained from the transcriptome dataset. **(C)** Functional characterization of sucrose transport activity by yeast complementation. The sucrose uptake‑deficient yeast strain SUSY7/ura3 was transformed with empty vector pDR196 (negative control), AtSUC2 (positive control), BnaA07.SUC2, or BnaC07.SUC2. Transformants were spotted on SD/-Ura medium containing sucrose as the sole carbon source. Yeast expressing *BnaA07.SUC2* or *BnaC07.SUC2* partially restored growth, whereas the empty vector did not. **(D)** Expression of *BnaA07.SUC2* in oilseed rape roots was analyzed by qPCR at 2, 4, 7, 14, 21, and 28 dpi with *P. brassicae*, using uninoculated roots at the corresponding time points as controls. Data are presented as means ± SD (n = 3). *P < 0.05 (two-way ANOVA followed by Tukey’s test).

To identify sucrose transporters involved in mediating sugar partitioning between *P. brassicae* and the host, whole transcriptome sequencing was conducted on oilseed rape roots following *P. brassicae* infection. In analyzing different time points (2–28 dpi), we identified *SUC* genes that were differentially expressed between 21 and 28 dpi compared with those in the control. The transcriptome data revealed a total of 17 *SUC* genes among the differentially expressed genes (DEGs) (S1 Data). Phylogenetic analysis using the nine *SUC* genes from *A. thaliana* as a reference classified these 17 genes into three types (Fig 2A). Type I is eudicot-specific and comprises ten oilseed rape *SUC* genes along with the *A. thaliana SUC* genes, including *AtSUC1* (AT1G71880), *AtSUC5* (AT1G71890), *AtSUC2* (AT1G22710), *AtSUC8* (AT2G14670), *AtSUC9* (AT5G06170), *AtSUC6* (AT5G43610), and *AtSUC7* (AT1G66570) [35,36]. Members of this clade localize to the plasma membrane and function in high-affinity sucrose uptake for phloem loading [37]. Type II is subdivided into Type IIA and Type IIB. Type IIB is monocot-specific. In contrast, Type IIA is present in both eudicots and monocots, and includes three oilseed rape *SUC* genes as well as *AtSUC3* (AT2G02860) [37,38]. Type III contains *SUC* genes from both monocots and eudicots, including four oilseed rape *SUC* genes as well as *AtSUC4* (AT1G09960), and functions in sucrose release from vacuoles [37–40]. Within this phylogenetic tree, *BnaC07.SUC2* forms a well-supported clade with *AtSUC2*, indicating that *BnaC07.SUC2* belongs to Type I and is orthologous to *AtSUC2*. Expression profiling revealed that these genes exhibited distinct temporal patterns and could be divided into early- and late-induced groups (Fig 2B). We focused on transporter genes that were highly expressed at 21 and 28 dpi relative to control and selected six candidates for further analysis (Fig 2B). Further analysis of the transcripts per million (TPM) values for these six genes at 21 and 28 dpi showed that the expression levels of *BnaA07.SUC2* and *BnaC07.SUC2* were significantly higher than those in the control at the same time points (S2A-B Fig). Therefore, these two genes were selected for subsequent functional validation in yeast.

To further characterize their function, *BnaA07.SUC2* and *BnaC07.SUC2* were heterologously expressed in the sucrose uptake-deficient yeast mutant SUSY7. Functional complementation assays showed that all transformants grew normally on glucose medium. However, when sucrose was provided as the sole carbon source, yeast expressing *BnaA07.SUC2* showed growth comparable to that of the positive control, whereas yeast expressing *BnaC07.SUC2* exhibited markedly weaker growth (Fig 2C). Thus, BnaA07.SUC2 has stronger transport activity than BnaC07.SUC2.

Quantitative real-time PCR (qPCR) analysis from 2 to 28 dpi revealed that *BnaA07.SUC2* transcript levels were low at 2 and 4 dpi, with no significant difference from the control. At 7 and 14 dpi, its expression was significantly downregulated relative to the control, whereas at 21 and 28 dpi, it was significantly upregulated (Fig 2D). This expression pattern is consistent with the characteristics of sucrose transporters identified as significantly upregulated at 21 and 28 dpi in this study. Therefore, *BnaA07.SUC2* was selected as a candidate gene involved in sucrose competition during the *P. brassicae-*oilseed rape interaction.

### Transport functional validation and localization analysis of BnaA07.SUC2

**Fig 3:**
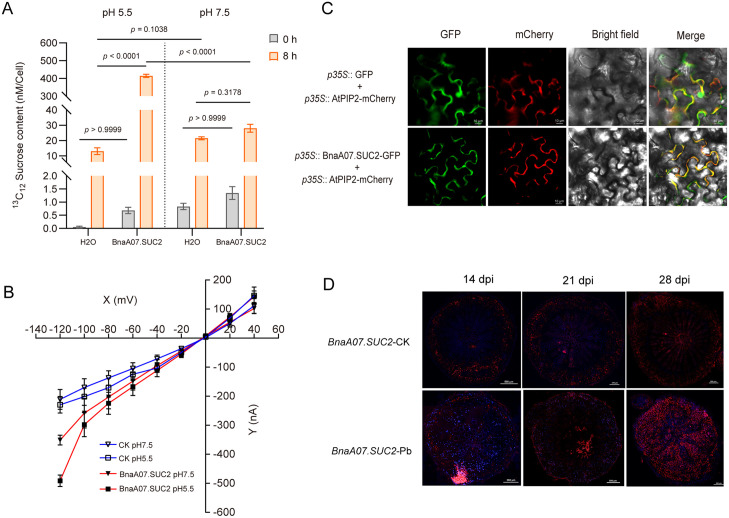
Characterization of BnaA07.SUC2 transport function and localization. **(A)** Sucrose uptake assay in *Xenopus laevis* oocytes expressing BnaA07.SUC2. Uptake was measured at pH 5.5 and pH 7.5 over a 0-8 h period. Water-injected oocytes served as negative control. Data represent means ± SD (n = 3). *P < 0.05 (two‑way ANOVA with Tukey’s test). **(B)** Current-voltage (I/V) relationship of BnaA07.SUC2‑mediated steady‑state currents. Currents were recorded at pH 5.5 or pH 7.5 using TEVC with voltage steps from +40 mV to –120 mV. Data are shown as means ± SE (n = 6). *P < 0.05 (one‑way ANOVA with Tukey’s test). **(C)** Subcellular localization of BnaA07.SUC2 in *Nicotiana benthamiana* epidermal cells. AtPIP2-mCherry was used as a plasma membrane marker. Scale bars = 10 µm. **(D)** Spatial expression pattern of *BnaA07.SUC2* transcripts in oilseed rape roots by FISH. Cross‑sections from *P. brassicae*-infected (Pb) and control (CK) roots were hybridized with Cy3‑labeled antisense probes. Nuclei were stained with DAPI. Scale bars = 200, 500 µm.

To further characterize its transport properties, *BnaA07.SUC2* was heterologously expressed in *Xenopus laevis* oocytes (S3A Fig). Uptake assays using [U-¹³C₁₂]-sucrose showed that at pH 5.5, oocytes expressing *BnaA07.SUC2* accumulated significantly more sucrose than those at pH 7.5. Furthermore, at pH 5.5, uptake was markedly higher than in water-injected controls, whereas no significant difference was observed at pH 7.5 (Fig 3A). These results indicate that BnaA07.SUC2 acts as a proton-coupled sucrose transporter. To further validate its function, we employed two-electrode voltage clamp (TEVC). At pH 5.5, oocytes expressing *BnaA07.SUC2* exhibited sustained sucrose-induced inward currents at holding potentials of -100 mV and -120 mV (Fig 3B), with the most pronounced current at -120 mV (S3B Fig). In contrast, no significant currents were detected at pH 7.5 (Fig 3B). Together, these data confirm that *BnaA07.SUC2* functions as a typical H ⁺ /sucrose symporter.

Subcellular localization analysis showed that the BnaA07.SUC2-GFP fusion protein colocalized with the plasma membrane marker AtPIP2A-mCherry in tobacco epidermal cells, confirming its plasma membrane localization (Fig 3C). To analyze its spatiotemporal expression during infection, we performed fluorescence in situ hybridization (FISH) for *BnaA07.SUC2* in oilseed rape roots at different stages of *P. brassicae* infection. In controls, transcripts were spatially restricted to the outer cell layers with low, stable abundance throughout the observation period (Fig 3D). Upon *P. brassicae* infection, expression was strongly induced in these outer layers and progressively redistributed into inner root tissues as infection progressed (Fig 3D). This suggests that *BnaA07.SUC2* expression is strongly induced upon *P. brassicae* infection, pointing to involvement in transmembrane sucrose transport during later stages of the host-pathogen interaction.

### *BnaA07.SUC2* enhances susceptibility by fueling *P. brassicae* with sucrose

To test the hypothesis that *BnaA07.SUC2* promotes susceptibility by supplying sucrose to the pathogen, we overexpressed this gene in the oilseed rape hairy root system and then inoculated the plants with *P. brassicae*. At 28 dpi, overexpression (OE) lines displayed pronounced swelling and accelerated spore maturation (S4A-B Fig). qPCR analysis confirmed that *BnaA07.SUC2* transcript levels were significantly higher in OE lines than in wild-type (WT) controls (S4C Fig). Disease quantification confirmed that OE lines had significantly increased swelling diameter and pathogen biomass compared with WT controls (S4D-E Fig).

**Fig 4:**
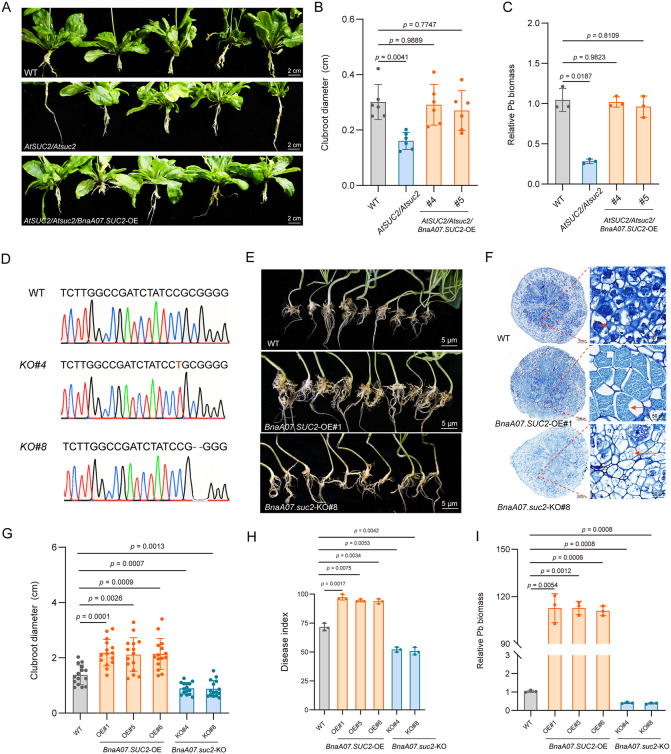
*BnaA07.SUC2* enhances host susceptibility. **(A)** Phenotypes of *A. thaliana* at 28 dpi: wild-type (WT, Col-0), the heterozygous *AtSUC2*/*Atsuc2* mutant, and the *AtSUC2*/*Atsuc2* mutant overexpressing (OE) *BnaA07.SUC2* (*AtSUC2*/*Atsuc2*/*BnaA07.SUC2-OE*). Scale bar = 2 cm. **(B)** Clubroot diameter of *A. thaliana* lines shown in **(A)**. Data are presented as mean ± SD (n = 6). *P < 0.05 (one-way ANOVA, Dunnett T3’s test). **(C)** Relative *P. brassicae* biomass at 28 dpi. *P. brassicae* biomass was assessed by qPCR and calculated using the 2^-ΔΔCT^ method. Data are presented as mean ± SD (n = 3). *P < 0.05 (one-way ANOVA, Dunnett T3’s). **(D)** Sequencing chromatograms of the edited sites in two independent knockout lines (*BnaA07.suc2*-KO) of oilseed rape (Westar). Mutation sites are highlighted in red. **(E)** Clubroot phenotypes of different oilseed rape lines at 28 dpi. *BnaA07.SUC2*-OE#1 and *BnaA07.suc2*-KO#8 lines. Scale bar = 5 cm. **(F)** Toluidine blue-stained paraffin cross sections showing pathogen proliferation within root cells in **(E)**. Scale bars: 500 μm (whole-section image), 50 μm (inset). Red arrows indicate *P. brassicae* within root cells. **(G)**-(I) Disease quantification of *BnaA07.SUC2*-OE and *BnaA07.suc2*-KO oilseed rape lines compared with WT at 28 dpi. **(G)** Clubroot diameter, **(H)** disease index, and **(I)** relative *P. brassicae* biomass. Data are presented as mean ± SD (n = 15 for diameter; n = 3 for disease index and biomass). *P < 0.05 (one-way ANOVA with Dunnett T3’s test).

To further investigate the function of *BnaA07.SUC2*, we performed complementation experiments using *A. thaliana* mutants. Because the homozygous *Atsuc2* mutant exhibits stunted growth and is unsuitable for genetic transformation, we used the heterozygous mutant (*AtSUC2*/*Atsuc2*) for functional complementation analysis. Following inoculation with *P. brassicae*, the *AtSUC2*/*Atsuc2* lines still displayed significantly reduced clubroot gall size compared with the WT (Fig 4A). We overexpressed *BnaA07.SUC2* in this mutant background, obtained two overexpression lines, and confirmed their expression levels (S5A Fig). At 28 dpi, both complementation lines showed significantly increased clubroot gall size compared with the mutant (Fig 4A). Statistical analysis revealed that clubroot diameter, pathogen biomass, and disease index were all significantly higher in the complementation lines than in the mutant (Fig 4B-C and S5B Fig).

We next generated stable *BnaA07.SUC2* OE lines in oilseed rape (S6A Fig). These lines accumulated significantly more sucrose than the WT (S6B Fig). This metabolic change was further supported by Fourier transform infrared (FTIR) spectroscopy, which revealed distinct carbohydrate-related spectral features in the *BnaA07.SUC2*-OE#1 line (S6C Fig), demonstrating that *BnaA07.SUC2* promotes sugar accumulation in cells.

To further determine the impact of *BnaA07.SUC2* on host susceptibility to clubroot disease, we generated knockout mutants in the Westar background. Sequencing of T_0_ plants revealed two lines, KO#4 and KO#8, harboring frameshift mutations in exon 1 (a 1 bp insertion and a 2 bp deletion, respectively) (Fig 4D). Upon *P. brassicae* inoculation, OE lines developed more severe clubroot symptoms and displayed accelerated spore maturation (Fig 4E-F), whereas KO mutants exhibited a strong clubroot resistance phenotype and a significant reduction in pathogen proliferation (Fig 4E-F). These observations were further supported by statistically significant increase in clubroot diameter, disease index, and pathogen biomass in the OE lines, and decrease in these parameters in the KO lines, compared with the WT (Fig 4G-I). Together, these results indicate that *BnaA07.SUC2* promotes host susceptibility by supplying sucrose to *P. brassicae*.

### BnaA05.MYC2 modulates disease susceptibility by downregulating *BnaA07.SUC2*

**Fig 5:**
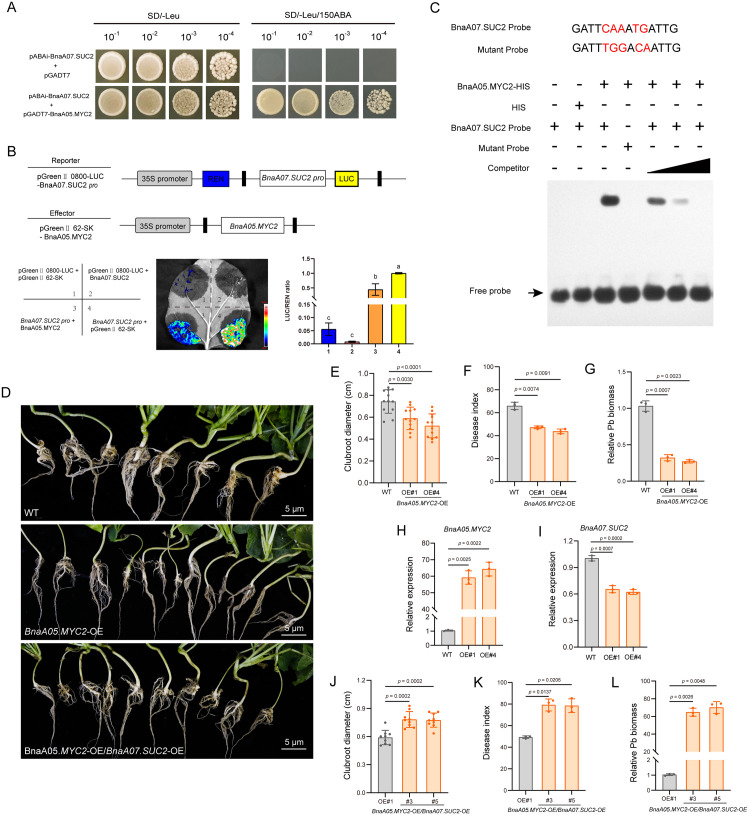
*BnaA05.MYC2* confers clubroot resistance via repressing *BnaA07.SUC2* expression. **(A)** A yeast one-hybrid assay showing the binding of BnaA05.MYC2 to the promoter of *BnaA07.SUC2*. **(B)** Dual-luciferase reporter assays investigating the regulatory relationship between BnaA05.MYC2 and the *BnaA07.SUC2* promoter. 1, 2, and 4 serve as controls. Data are presented as means ± SD (n = 3). *P < 0.05 (one-way ANOVA with Tukey’s test). **(C)** Electrophoretic mobility shift assays confirming direct binding of BnaA05.MYC2 protein to the *BnaA07.SUC2* promoter. Binding was competed by an excess of unlabeled wild-type probe (competitor), but not by mutant probe. Mutated nucleotides in the mutant probe are highlighted in red. “–” and “+” denote absence or presence of the indicated component **(D)** Clubroot phenotypes of oilseed rape at 28 dpi: WT, *BnaA05.MYC2*-OE line, and a hybrid line generated by crossing *BnaA05.MYC2*-OE#1 with *BnaA07.SUC2*-OE#1 (*BnaA05.MYC2*-OE/*BnaA07.SUC2*-OE). Scale bar = 5 cm. **(E)**-(G) Disease quantification of *BnaA05.MYC2*-OE oilseed rape lines compared with WT at 28 dpi. **(E)** Clubroot diameter, **(F)** disease index, and **(G)** relative *P. brassicae* biomass. Data are mean ± SD (n = 12 for diameter; n = 3 for disease index and biomass). *P < 0.05 (one-way ANOVA with Dunnett T3’s test). **(H)**-(I) Expression levels of **(H)** *BnaA05.MYC2* and **(I)** *BnaA07.SUC2* in two *BnaA05.MYC2*-OE oilseed rape lines relative to WT. Data are mean ± SD (n = 3). *P < 0.05 (one-way ANOVA with Dunnett T3’s test). **(J)**-(L) Disease quantification of *BnaA05.MYC2*-OE#1/*BnaA07.SUC2*-OE#1 oilseed rape lines compared with *BnaA05.MYC2-*OE#1 line at 28 dpi. **(J)** Clubroot diameter, **(K)** disease index and **(L)** relative *P. brassicae* biomass. Data are presented as means ± SD (n = 9 for diameter; n = 3 for disease index and biomass). *P < 0.05 (one-way ANOVA with Dunnett T3’s test).

To identify upstream regulators of *BnaA07.SUC2*, we conducted a yeast one-hybrid (Y1H) screen using the *BnaA07.SUC2* promoter as bait to screen a cDNA library derived from *P. brassicae*-oilseed rape interaction samples. This screen identified the transcription factor BnaA05.MYC2 as a putative regulator (Fig 5A). Dual-luciferase reporter (Dual-LUC) assays further showed that BnaA05.MYC2 significantly repressed the transcriptional activity of the *BnaA07.SUC2* promoter (Fig 5B). Consistently, electrophoretic mobility shift assays (EMSA) confirmed that BnaA05.MYC2 protein binds directly to a specific MYC-binding element within the *BnaA07.SUC2* promoter, located between positions -657 and -652 bp (Fig 5C). Collectively, these findings establish BnaA05.MYC2 as a direct transcriptional repressor of *BnaA07.SUC2*.

To validate the role of *BnaA05.MYC2* in clubroot disease, we performed complementation experiments using the homozygous *myc2* mutant in *A. thaliana*. At 28 dpi, the *myc2* mutant exhibited significantly enhanced susceptibility to *P. brassicae*, with clubroot gall size markedly larger than that of the WT (S7A Fig). Overexpression of *BnaA05.MYC2* in the *myc2* mutant resulted in substantially increased *BnaA05.MYC2* transcript levels (S7B Fig). At 28 dpi with *P. brassicae*, these plants exhibited reduced disease susceptibility compared with the mutant (S7A Fig), as evidenced by significant decreases in clubroot diameter, disease index, and *P. brassicae* biomass (S7C-E Fig).

To further confirm its function, we generated *BnaA05.MYC2* OE lines in oilseed rape. At 28 dpi, OE lines exhibited a strong resistance phenotype compared with the WT (Fig 5D), characterized by smaller clubroot diameter, a lower disease index and reduced *P. brassicae* biomass (Fig 5E-G). Together, these results indicate that *BnaA05.MYC2* enhances clubroot resistance in host. Gene expression analysis revealed that *BnaA05.MYC2* OE lines had significantly elevated transcript levels of *BnaA05.MYC2*, whereas *BnaA07.SUC2* expression was markedly reduced (Fig 5H-I). This result indicates that *BnaA07.SUC2* is substantially suppressed upon *BnaA05.MYC2* overexpression, which is consistent with *BnaA05.MYC2* acting as a negative regulator of *BnaA07.SUC2*. These findings are in agreement with the Dual-LUC assay results (Fig 5B).

To investigate whether the clubroot resistance conferred by *BnaA05.MYC2* depends on the suppression of *BnaA07.SUC2*, we generated co-expression lines by crossing *BnaA05.MYC2* OE lines with *BnaA07.SUC2* OE lines in oilseed rape. At 28 dpi, the co-expression lines showed markedly increased susceptibility compared with the *BnaA05.MYC2* OE line (Fig 5D), with significantly increased clubroot diameter, disease index, and pathogen biomass relative to the *BnaA05.MYC2* OE line (Fig 5J-L). Taken together, these results indicate that BnaA05.MYC2 enhances resistance to *P. brassicae* by transcriptionally repressing *BnaA07.SUC2*.

### *BnaA07.SUC2* expression was regulated by host jasmonate signaling during *P. brassicae* infection

**Fig 6:**
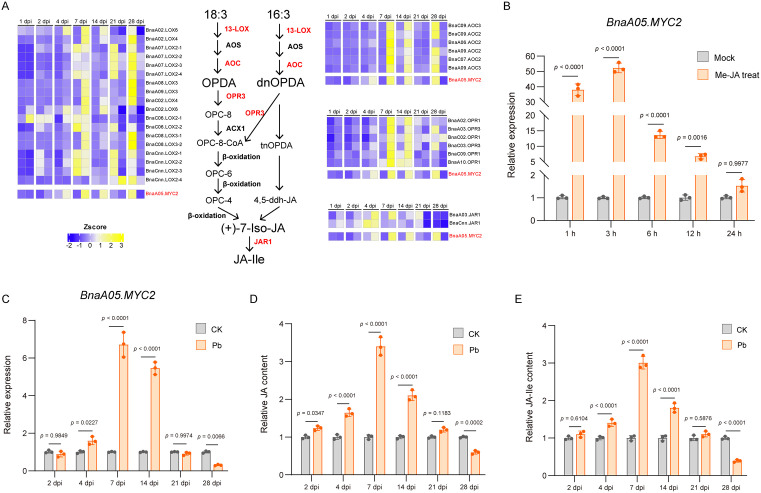
*BnaA05.MYC2* was regulated by JA signaling during *P. brassicae* infection. **(A)** Heatmap showing the expression profiles of key JA/JA-Ile biosynthetic genes in oilseed rape roots at different time points after infection. For each time point, the two columns represent the control (left) and *P. brassicae*-inoculated (right) samples. The expression profile of *BnaA05.MYC2* (in red) is shown below the heatmap. Data were derived from transcriptome sequencing of *P. brassicae*-infected oilseed rape roots. **(B)** Expression of *BnaA05.MYC2* in oilseed rape root tissues following MeJA treatment relative to untreated controls at the corresponding time points. Data are presented as mean ± SD (n = 3). *P < 0.05 (two‑way ANOVA with Tukey’s test). **(C)** qPCR analysis of *BnaA05.MYC2* expression in oilseed rape roots at different time points after *P. brassicae* infection, relative to uninoculated controls at the corresponding time points. Data are presented as mean ± SD (n = 3). *P < 0.05 (two-way ANOVA with Tukey’s test). **(D)**-(E) Concentrations of **(D)** JA and **(E)** JA-Ile in inoculated oilseed rape roots relative to uninoculated controls at the corresponding time points, quantified by UPLC‑MS/MS. Data are presented as mean ± SD (n = 3). *P < 0.05 (two-way ANOVA with Tukey’s test).

Given that MYC2 transcription factors are key mediators of downstream responses in the JA signaling pathway [24,26,41], we hypothesized that *P. brassicae* infection affects the JA signaling pathway in oilseed rape, thereby influencing *BnaA05.MYC2* expression. To test this hypothesis, we analyzed the expression patterns of *BnaA05.MYC2* and JA signaling pathway genes during infection using transcriptome sequencing data from oilseed rape roots at different time points (2–28 dpi). The results revealed that JA biosynthesis-related genes (*13-LOX*, *AOC*, *OPR*, and *JAR*) were induced during the early stages of infection (7–14 dpi), with expression levels decreasing thereafter (21–28 dpi) (Fig 6A and S2 Data). The expression pattern of *BnaA05.MYC2* was consistent with that of the JA biosynthesis genes (Fig 6A and S2 Data). These results indicated that *BnaA05.MYC2* expression is likely regulated by JA signaling.

To further validate this regulatory relationship between *BnaA05.MYC2* and JA, we treated five-leaf-stage oilseed rape seedlings with methyl jasmonate (MeJA) via root drench and examined *BnaA05.MYC2* expression levels in the roots at different time points after treatment. The results showed that the transcript level increased significantly at 1 h post-treatment, peaked at 3 h, and then gradually declined (Fig 6B). These findings confirm that JA signaling positively regulates *BnaA05.MYC2* expression.

Furthermore, we examined *BnaA05.MYC2* expression in oilseed rape roots at different time points after *P. brassicae* inoculation by qPCR. The qPCR results were consistent with the transcriptome data (Fig 6A and S2 Data): expression levels were significantly higher than those in non-inoculated controls at 7 and 14 dpi, but significantly lower at 21 and 28 dpi (Fig 6C). To further examine the dynamic changes of the JA signaling pathway in oilseed rape roots after *P. brassicae* infection, we measured the levels of JA and its bioactive form, jasmonoyl-isoleucine (JA-Ile), at different time points post-inoculation. The results showed that the accumulation patterns of JA and JA-Ile were highly consistent with the expression pattern of *BnaA05.MYC2* (Fig 6C): both were significantly higher than those in the non-inoculated controls at 7 and 14 dpi, but significantly lower at 21 and 28 dpi (Fig 6D-E).

Collectively, these results demonstrate that the JA signaling pathway in oilseed rape roots is dynamically regulated during *P. brassicae* infection. At the later stage of infection (21 and 28 dpi), JA signaling is suppressed, leading to decreased *BnaA05.MYC2* expression and a significant increase in *BnaA07.SUC2* expression.

**Fig 7:**
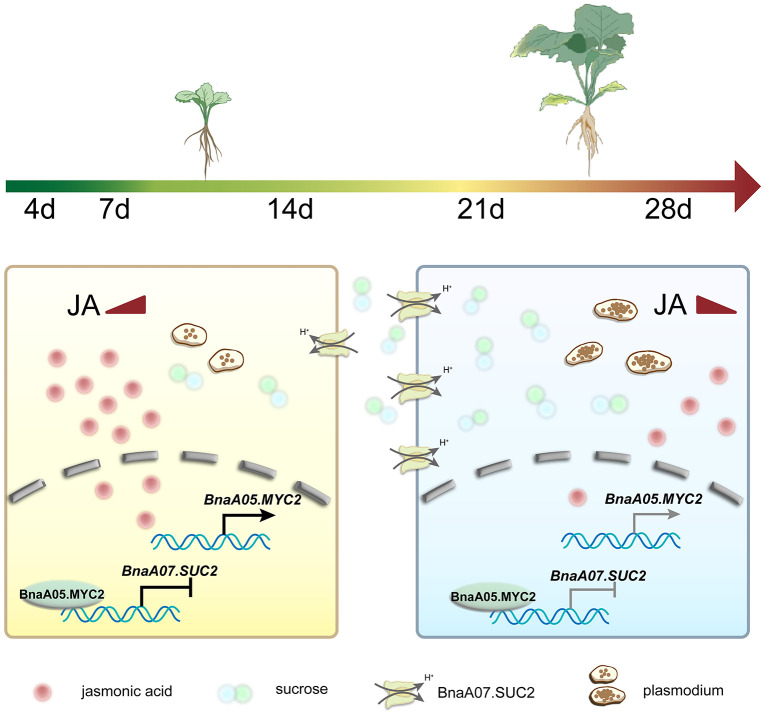
A working model of JA-mediated regulatory of *BnaA07.SUC2* during *P. brassicae* infection. The model depicts the molecular events at two distinct stages: the early phase (before 14 dpi) and the late phase (after 14 dpi). Early phase (before 14 dpi): Upon infection with *P. brassicae*, the JA signaling pathway is activated, leading to elevated levels of JA and JA-Ile. This upregulates the expression of *BnaA05.MYC2*, which in turn represses *BnaA07.SUC2* expression. Late phase (after 14 dpi): The JA signaling pathway is suppressed, accompanied by reduced levels of JA and JA-Ile. Consequently, *BnaA05.MYC2* expression declines, which attenuates its repressive effect on *BnaA07.SUC2*. This results in significant upregulation of *BnaA07.SUC2*, facilitating sugar uptake and promoting the proliferation of *P. brassicae*. Black flat arrows indicate enhanced inhibitory effects, gray flat arrows represent weakened inhibitory effects, black pointed arrows indicate enhanced promoting effects, and gray pointed arrows represent weakened promoting effects.

Based on these findings, we propose a working model for the regulation of *BnaA07.SUC2* during *P. brassicae* infection in oilseed rape (Fig 7). During the early infection stage, the host JA pathway is activated, leading to the accumulation of JA and JA-Ile. This induces expression of the transcription factor *BnaA05.MYC2*, which then directly represses *BnaA07.SUC2* by binding to its promoter, resulting in its initial downregulation. During the later infection stage, with disease progression and increased pathogen biomass, the JA pathway is suppressed, JA and JA-Ile levels decline, and *BnaA05.MYC2* expression decreases. This attenuates BnaA05.MYC2-mediated repression and leads to derepression of *BnaA07.SUC2*, resulting in its significant upregulation during the later stage. Consequently, host carbon resources are redirected to fuel pathogen proliferation.

## Discussion

In plants, SUC2 is a multifunctional transporter essential for phloem loading and source-sink translocation [42], abiotic stress responses [6,43,44], and apoplastic sucrose retrieval in immunity [45]. Its potential role in energy resource competition during pathogen-plant interactions remains unclear.

Our findings demonstrate that the expression of *BnaA07.SUC2* is significantly upregulated during the later stages of infection (21 and 28 dpi) (Fig 2B-D). Microscopic examination of root cross-sections revealed that this stage corresponds to the massive proliferation phase of *P. brassicae* within host cells (Fig 1B and S1 Fig). The observed temporal correlation strongly suggests that the induced *BnaA07.SUC2* activity functions to supply carbon sources, thereby supporting this high energy demand growth phase of *P. brassicae*. FISH revealed that *P. brassicae* infection induces *BnaA07.SUC2* expression in roots, redirecting sucrose flow from the apoplast toward infected cells (Fig 3). Genetic experiments in both oilseed rape and *A. thaliana* confirmed that elevated *BnaA07.SUC2* expression is associated with increased host susceptibility (Fig 4). These results indicate that this apoplastic sucrose supply pathway is crucial for carbon acquisition by *P. brassicae.* The strong carbon demand in infected roots is likely to enhance rootward carbon allocation in the host, accompanied by the upregulation of *SUC* genes in source tissues to promote phloem loading [3,46–48]. Together, these coordinated responses establish a systemic metabolic reprogramming that benefits the pathogen.

As a protist pathogen, *P. brassicae* exists as a multinucleate plasmodium within the host cytoplasm. Its nutrient acquisition strategies are multifaceted, including the manipulation of host carbon and nitrogen allocation, phloem transport, and long-distance signaling to meet its nutritional demands, as well as the direct uptake of small solutes via transmembrane transporters and the phagocytosis of larger host-derived macromolecules [48–50]. Following the massive influx of sucrose into infected cells mediated by host transporters, the pathogen likely imports these sugars through its own transporters to support rapid proliferation [50]. Therefore, identifying and functionally characterizing sugar transporters on the *P. brassicae* plasma membrane represents a critical next step to systematically elucidate how the pathogen directly acquires carbon from the host cytoplasm. Furthermore, *P. brassicae* infection induces substantial starch granule accumulation in host cells [51]. This observation suggests that the pathogen manipulates host carbon metabolism to promote starch synthesis, followed by the phagocytic internalization of these granules for storage and subsequent utilization as an energy reserve.

It is well established that the jasmonic acid (JA) signaling pathway positively regulates resistance to clubroot disease [52]. This study demonstrates that during *P. brassicae* infection, the expression of *BnaA07.SUC2* is regulated by BnaA05.MYC2 through JA signaling. While the restoration of the clubroot phenotype upon co-overexpression of *BnaA05.MYC2* and *BnaA07.SUC2* strongly supports the hypothesis that MYC2-mediated resistance to clubroot is attributable, at least in part, to the repression of *SUC2*, this mechanism likely represents only one facet of a broader resistance strategy. Indeed, it is important to acknowledge the multifaceted roles of MYC2 in plant immunity, which extend beyond sugar transport regulation and may involve additional layers of defense. For instance, in citrus, the PUB21-MYC2 module regulates resistance to Huanglongbing by modulating MYC2 protein stability; the stabilization of MYC2 activates multiple defense proteins and secondary metabolite biosynthetic pathways, conferring high resistance to the pathogen [53]. In *A. thaliana*, MYC2 also induces the expression of genes related to defense signaling, including RPM1-interacting protein 4 (RIN4), Mitogen-activated protein kinase kinase 4 (MKK4), by binding to their promoters and regulating pathogen-mediated immunity [54]. Furthermore, studies in other plant species have demonstrated that MYC2 regulates the biosynthesis of defensive secondary metabolites such as glucosinolates [55], and mediates crosstalk between jasmonate and salicylate signaling pathways [56]. Therefore, the observed resistance to clubroot in *BnaA05.MYC2* overexpression lines may reflect the combined effects of altered carbon partitioning, compromised chemical defense, and dysregulation of broader hormonal networks. The complementation assay with *BnaA07.SUC2* overexpression specifically restores the sugar transport-related function, but other MYC2-regulated processes likely remain affected, which could explain the partial or context-dependent nature of the phenotype.

This study uncovers a novel role for a SUC transporter, BnaA07.SUC2, in the pathogenesis of *P. brassicae* and identifies its upstream regulator, BnaA05.MYC2. These findings not only advance our fundamental understanding of host-pathogen interactions but also provide a potential genetic target for the development of clubroot resistance.

## Materials and Methods

### Plant materials and growth conditions

The oilseed rape (*Brassica napus* L.) materials used in this study were all of the cultivar ‘Westar’, while all *Arabidopsis thaliana* plants were in the Columbia-0 (Col-0) background. Prior to sowing, oilseed rape or *A. thaliana* seeds were stratified at 4°C in darkness for 2 days. Plants were cultivated in a controlled greenhouse environment under a 16-h light/8-h dark photoperiod with a light intensity of 300 μmol m ⁻ ² s ⁻ ¹. Daytime and nighttime temperatures were maintained at 22 ± 2°C and 18 ± 2°C, respectively, with a relative humidity of 60%. A sterilized potting mix consisting of peat, vermiculite, and perlite in a 3:1:1 (v/v/v) ratio was used. Hairy roots were cultured in a growth chamber maintained under constant darkness, with the same temperature and humidity conditions as described above.

The *A. thaliana* mutants used in this study, *Atsuc2* (SALK_038124), *Atmyc2* (SALK_017005C) were obtained from the Arabidopsis Biological Resource Center (ABRC, http://www.arabidopsis.org/abrc/) and conﬁrmed with T-DNA border primers and gene-speciﬁc primers (S3 Data). Heterozygous *AtSUC2/Atsuc2* mutants were identified by three-primer PCR and selected for genetic transformation. Homozygous *Atmyc2* mutants were similarly identified and used for transformation at the flowering stage.

### Isolation of *P. brassicae* resting spores

Resting spores of *P. brassicae* were isolated from clubroot galls harvested from infected oilseed rape plants in Sichuan Province, China. Clubroot galls were homogenized and filtered through eight layers of gauze, and then centrifuged at 3,100 rpm for 15 min, and the supernatant was discarded. The pellet was resuspended in sterile distilled water three times. To remove residual plant debris, the pellet was resuspended in 10 mL of filter-sterilized 50% (w/v) sucrose solution and centrifuged at 3,100 rpm for 10 min. The supernatant, along with the grayish upper layer of the pellet containing the resting spores, was carefully transferred to a new sterile 50 mL centrifuge tube, while the lower debris was discarded. The spore pellet was washed twice to remove residual sucrose. The concentration of purified resting spores was determined using a hemocytometer. Finally, the spore suspension was diluted with sterile distilled water to a final concentration of 1 × 10^6^ spores·mL ⁻ ¹.

### Surface sterilization of *P. brassicae* resting spores

The resting spore suspension was centrifuged at 3,100 rpm for 15 min, and the supernatant was discarded. The pellet was resuspended in freshly prepared 2% (w/v) chloramine-T solution and gently agitated at room temperature for 20 min. The suspension was then centrifuged at 3,100 rpm for 10 min, and the supernatant was removed. The pellet was washed three times with sterile distilled water. Following the final wash, the pellet was resuspended in an antibiotic solution containing 1,000 µg/mL colistin sulfate, 1,000 µg/mL vancomycin hydrochloride, and 6,000 µg/mL cefotaxime sodium, and incubated overnight. After incubation, the spores were washed three times with sterile distilled water to remove residual antibiotics. The final pellet was resuspended in sterile distilled water containing cefotaxime sodium (200 µg/mL) and stored at 4°C for further use.

### Inoculation with *P. brassicae* resting spores

For inoculation of soil-grown plants: five-leaf-stage oilseed rape or *A. thaliana* was selected for inoculation. The soil around the roots was gently pushed aside, and 1 mL of freshly isolated *P. brassicae* resting spore suspension (1 × 10⁶ spores·mL ⁻ ¹) was injected into the rhizosphere. The soil was then carefully backfilled to cover the roots. Inoculated plants were maintained in a greenhouse under normal growth conditions.

Inoculation of hairy roots: under sterile conditions, root segments (approximately 3 cm in length) were excised from cultured hairy roots and placed on MS solid medium (3% sucrose, pH 5.8, 0.5% agar). After one day of culture, when root hairs had emerged and the roots had established contact with the medium, 1 mL of surface-sterilized *P. brassicae* resting spore suspension (1 × 10⁶ spores·mL ⁻ ¹) was evenly applied to each plate, ensuring complete contact with all root segments. The plates were then sealed and incubated in a growth chamber.

### Cytological observation of *P. brassicae* infection

To investigate the colonization and distribution of *P. brassicae*, root samples were collected from both inoculated hairy roots and oilseed rape plants. Hairy root samples were harvested at 28 days post-inoculation (dpi), while oilseed rape clubroot tissues were collected at 14, 21, and 28 dpi to monitor the progression of infection. All samples were fixed in Formalin-Aceto-Alcohol (FAA) solution, dehydrated through an ethanol series, cleared in xylene, and embedded in paraffin. Transverse sections (5 μm thickness) were cut using a rotary microtome (Leica RM2016), stained with 0.1% (w/v) toluidine blue, and mounted with coverslips. The sections were then observed under a light microscope (Nikon Eclipse Ci) to assess the distribution of resting spores or plasmodia within the host tissues.

### CFDA labelling

The susceptible oilseed rape cultivar Westar was used to examine the assimilate transport route in the root. A 1-cm segment from the mature zone of the root was exposed by carefully removing the surrounding surface soil and was rinsed with distilled water. The exposed root segment was then gently abraded with a blade to disrupt the epidermis followed by rinsing with distilled water. To inhibit wound-induced callose deposition and sieve tube blockage, the treated area was wrapped with absorbent cotton soaked in 2.5 mmol·L ⁻ ¹ ethylenediaminetetraacetic acid (EDTA) solution for 40 min. The cotton was then replaced with a fresh piece, and 200 μL of ice-cold 1 mg·mL ⁻ ¹ 5(6)-carboxyfluorescein diacetate (CFDA) solution was applied. The site was sealed with Parafilm to prevent evaporation. To mitigate fluorescence quenching, the sealed area was wrapped in aluminum foil for light protection. The exposed root was then carefully covered with soil to maintain the rhizosphere environment, and the plants were returned to normal growing conditions for 48 h. After incubation, the CFDA-loaded root segment was excised, rinsed, and immediately sectioned by freehand sectioning. Sections were observed promptly under a confocal laser scanning microscope (Olympus FV3000-IX83) using 488-nm laser excitation for CFDA fluorescence detection.

### Transcriptome analysis

For transcriptome analysis, the roots of oilseed rape seedlings at the five-leaf stage were inoculated with *P. brassicae* and were collected at 2, 4, 7, 14, 21, and 28 dpi, alongside non-inoculated controls at the corresponding time points. Samples were ground to powder in liquid nitrogen, and total RNA was isolated using TRNzol reagent (DP405–02, TIANGEN, Beijing, China) according to the manufacturer’s protocol. Library construction and sequencing were performed by Wuhan Metware Biotechnology Co., Ltd. (Wuhan, China) on an Illumina platform, generating 150-bp paired-end reads. Transcriptome profiling was performed as described previously [57]. Briefly, raw reads obtained from the Illumina sequencing platform were quality-filtered using Fastp (v.0.23.2). The clean reads were aligned to the oilseed rape reference genome (v.4.1) using Hisat2 (v.2.2.1), then normalized to transcripts per million (TPM) using StringTie (v.2.1.6). Differential expression analysis was conducted with DESeq2 (v.1.22.1). Genes with |log₂FC| ≥ 1 and an adjusted *P*-value < 0.05 (Benjamini & Hochberg correction) were considered differentially expressed.

### Yeast complementation assay

Based on the CDS sequences of *BnaA07.SUC2* (BnaA07g10320D) and *BnaC07.SUC2* (BnaC07g13570D) obtained from the *B. napus* multi-omics information resource BnIR (http://yanglab.hzau.edu.cn/BnIR), and that of the positive control *AtSUC2* (AT1G22710) from the TAIR database (https://www.arabidopsis.org), gene-specific primers were designed using Primer Premier 5 software. The coding sequences of *BnaA07.SUC2* and *BnaC07.SUC2* were amplified from ‘Westar’ cDNA, while *AtSUC2* was amplified from *A. thaliana* (Col-0) cDNA. The target fragments were gel-purified using the EasyPure Quick Gel Extraction Kit (EG101–01; TransGen Biotech, Beijing, China) and ligated into the pEASY-T5 Zero Cloning Vector (CT101–01; TransGen Biotech) with T4 DNA ligase (FL101–01; TransGen Biotech). The ligation products were transformed into *Escherichia coli* DH5α competent cells (CD201–01; TransGen Biotech). After recovery, the cell suspensions were plated on Lysogeny Broth (LB) agar containing ampicillin (50 μg/mL) and incubated inverted at 37°C for 12 h. Positive colonies were confirmed by colony PCR using M13F/R primers and subsequently verified by sequencing (Sangon Biotech, Shanghai, China). The resulting verified plasmids were designated as T-*A07.SUC2*, T-*C07.SUC2*, and T-*AtSUC2*. Primer sequences are listed in S3 Data.

Using the three recombinant T-vectors as templates, PCR amplification was performed with gene-specific primers introducing *Spe*I and *Xho*I restriction sites (S3 Data). The purified PCR products and the pDR196 vector were separately digested with *Spe*I (JN201–01; TransGen Biotech) and *Xho*I (JX201–01; TransGen Biotech). The digested fragments were gel-purified using the EasyPure Quick Gel Extraction Kit (EG101–01; TransGen Biotech), and each gene fragment was ligated into the linearized pDR196 vector at a molar ratio of 3:1 using T4 DNA ligase (FL101–01; TransGen Biotech) at 16°C for 1 h. These constructs, designated pDR196‑*A07.SUC2*, pDR196‑*C07.SUC2*, and pDR196‑*AtSUC2*, place the respective genes under the control of the PMA promoter. The ligation mixtures were transformed into *E. coli* DH5α competent cells (CD201–01; TransGen Biotech) and plated on LB agar containing ampicillin (50 μg/mL). Positive colonies were screened by colony PCR using pDR196-F/R primers and further confirmed by sequencing (Sangon Biotech, Shanghai, China). The constructs with the correct sequences were used for subsequent experiments. Primer sequences are listed in S3 Data.

The *Saccharomyces cerevisiae* mutant strain SUSY7/ura3, which lacks extracellular sucrose invertase activity, was used for functional complementation assays. The three recombinant plasmids, along with the empty pDR196 vector as a negative control, were transformed into yeast mutant competent cells using the lithium acetate method. Transformants were selected on SD‑Ura agar plates (pH 5.8) containing 2% glucose and incubated at 30°C for 2–3 days until colonies appeared. Single colonies were inoculated into YPD liquid medium containing 2% glucose and cultured overnight at 30°C with shaking. Plasmids were extracted using a Yeast Plasmid Extraction Kit (TIANGEN, Beijing, China) according to the manufacturer’s instructions. The presence of the target plasmids was confirmed by PCR using the pDR196‑F/R primers.

For the complementation assay, positive yeast transformants were cultured in liquid SD‑Ura medium containing 2% glucose until the OD600 reached 0.6. The cultures were then serially diluted (10 ⁻ ¹, 10 ⁻ ², 10 ⁻ ³, and 10 ⁻ ⁴) with sterile SD‑Ura medium containing 2% sucrose. Aliquots (5 μL) of each dilution were spotted onto SD‑Ura solid medium supplemented with either 2% glucose or 2% sucrose as the sole carbon source. The plates were incubated at 30°C for 3–4 days, and growth phenotypes were observed and documented.

### Gene functions validation in *X. laevis* oocytes

The coding sequence of *BnaA07.SUC2* was amplified by PCR using the plasmid T-*A07.SUC2* as a template, with primers containing *Sma*I and *Eco*RI restriction sites. The purified PCR product was then subcloned into the pGEMHE vector downstream of the T7 promoter using a double digestion and ligation method, following the same procedure as described for the construction of pDR196-*A07.SUC2*. Positive clones were identified by PCR using the primers pGEMHE-F/R, and the resulting recombinant plasmid was designated pGEMHE-*SUC2*. Primer sequences are listed in S3 Data. Capped cRNA was synthesized in vitro from linearized plasmid DNA using the T7 mMessage mMachine kit (Invitrogen). Stage IV-VI X. laevis oocytes were injected with 25 nL of cRNA (experimental group) or nuclease-free water (control group). Injected oocytes were maintained at 16°C in Ringer’s buffer for 36 h to permit protein expression and trafficking. Subcellular localization was subsequently examined by confocal laser scanning microscopy (Leica SP8) with the target protein fused to mCherry (excitation 561 nm, emission 580–680 nm).

The sugar uptake assay was performed as previously described with modifications [58]. Post-injection, oocytes were incubated for 1 min in Ringer’s (96 mM NaCl, 2 mM KCl, 1 mM CaCl2, 1 mM MgCl_2_, 5 mM Hydroxyethylpiperazine Ethane Sulfonic Acid (HEPES), 10 mM sorbitol, pH was adjusted to 7.5 with NaOH) buffer containing 5 mM [U-¹³C₁₂]-sucrose with gentle agitation, followed by a brief rinse. Oocytes were then incubated in fresh uptake buffer (pH 5.5 or 7.5) containing 5 mM ¹³C₁₂-sucrose at 18°C for 2–8 h. Following the uptake period, groups of three oocytes were pooled as one biological replicate, homogenized, and the intracellular ¹³C₁₂‑sucrose content was quantified by liquid chromatography‑tandem mass spectrometry (LC‑MS/MS; AB SCIEX API 6500).

Two-electrode voltage-clamp (TEVC) recordings were performed on stage V-VI *X. laevis* oocytes. Oocytes were maintained overnight at 18°C in Ringer’s solution prior to injection. Each oocyte was injected with 30 ng of in vitro-transcribed cRNA or an equivalent volume of nuclease-free water (control) and then incubated in the same solution for two days to allow protein expression. For recording, oocytes were perfused with a bath solution containing 30 mM sucrose, 5 mM NaCl, 4 mM KCl, 1 mM MgCl₂, 10 mM HEPES, with pH adjusted to 7.5 or 5.5 using NaOH/HCl, and osmolality adjusted to 220 mOsm/L with sorbitol. The pipette solution contained 3 M KCl. Membrane currents were recorded using an Axoclamp 900A amplifier. The voltage protocol consisted of a 0.1-s prepulse to -40 mV, followed by test pulses from +40 to -120 mV in -20 mV steps (1.5 s duration each), and a final 0.5-s deactivation step at -40 mV.

### Subcellular localization

The coding sequence of *BnaA07.SUC2* was amplified by PCR using the plasmid T-*A07.SUC2* as a template, with primers containing *Xba*I *and* *Kpn*I restriction sites. The purified PCR product was then subcloned into the pCAMBIA121 vector downstream of the CaMV 35S promoter using a double digestion and ligation method. Positive clones were identified by PCR using primers pBI121-F/R, and the resulting recombinant plasmid was designated pBI121-*SUC2*. Primer sequences are listed in S3 Data.

The expression vector pBI121-*SUC2* was transformed into *Agrobacterium tumefaciens* strain GV3101 competent cells using the freeze-thaw method. The transformation mixture was spread onto LB solid medium containing 50 μg/mL kanamycin and 20 μg/mL rifampicin, and incubated inverted at 28°C for 2–3 days. Single colonies were selected and inoculated into LB liquid medium supplemented with the corresponding antibiotics. Positive transformants were identified by colony PCR using the primer pair pBI121-F/R, and the confirmed strains were preserved for further use.

For subcellular localization analysis, the recombinant vector pBI121-*BnaA07.SUC2*-eGFP was transiently expressed in *Nicotiana benthamiana* leaves via *A. tumefaciens*-mediated infiltration, following the method described by Sparkes et al., [59]. *A. tumefaciens* cultures harboring pBI121-*BnaA07.SUC2*-eGFP or the plasma membrane marker were separately grown in 10 mL LB liquid medium containing 50 μg/mL kanamycin and 50 μg/mL rifampicin at 28°C with shaking (220 rpm) until OD_600_ reached 1.0. Cells were harvested by centrifugation (8,000 rpm, 5 min, room temperature) and resuspended in infiltration buffer (10 mM MgCl₂, 10 mM MES, pH 5.6 with KOH, 0.2 mM acetosyringone) to a final OD_600_ of 0.8-1.0. The suspensions were incubated in the dark at room temperature for 2 h prior to infiltration.

For infiltration, the bacterial suspension was gently injected into the abaxial side of leaves from 4- to 6-week-old *N. benthamiana* plants using a 1 mL needleless syringe, with multiple infiltration sites per leaf. Excess bacterial solution was gently removed from the leaf surface with absorbent paper. Infiltrated plants were kept in the dark for 12 h and then transferred to normal light conditions for an additional 2–3 days. Fluorescence signals were examined at 72 h post-infiltration using a Leica DM4B fluorescence microscope with the following filter sets: GFP (excitation 488 nm, emission 495–545 nm) and mCherry (excitation 561 nm, emission 580–680 nm).

### ISH and FISH

Root segments (3–5 mm) from inoculated or control oilseed rape plants were fixed in FAA overnight, dehydrated through a graded ethanol series, and embedded in paraffin. Transverse sections (8–10 μm thick) were cut using a rotary microtome (Leica RM2016). Gene‑specific DNA probes (S3 Data) were labeled with digoxigenin (DIG) and hybridized to target sequences in the sections. Subsequently, sections were incubated with alkaline phosphatase (AP)‑conjugated anti‑DIG antibodies. Hybridization signals were developed using BCIP/NBT as the chromogenic substrate (Boster). Stained sections were imaged under an upright light microscope (Nikon Eclipse Ci).

For FISH, Cy3‑labeled oligonucleotide probes targeting specific gene sequences were used. Following hybridization, nuclei were counterstained with DAPI (4′,6‑diamidino‑2‑phenylindole). Fluorescence imaging was performed using an upright fluorescence microscope (Leica DM4 B) with the following filter sets: DAPI (excitation 330–380 nm, emission 420 nm) and Cy3 (excitation 510–560 nm, emission 590 nm).

### Overexpression vector construction

The coding sequence of *BnaA05.MYC2* (BnaA05g18020D) was amplified using gene-specific primers (primer sequences are listed in S3 Data) and cloned into the pEASY-T5 Zero Cloning Vector (CT101–01; TransGen Biotech) to generate the T-*MYC2* vector, following the same method used for the construction of T-*A07.SUC2*.

The coding sequences of *BnaA07.SUC2* and *BnaA05.MYC2* were amplified by PCR using the plasmids T-*A07.SUC2* and T-*MYC2* as templates, respectively, with primers containing *Kpn*I and *Sal*I restriction sites. The purified PCR products were then subcloned into the pCAMBIA1300s vector downstream of the CaMV 35S promoter using a double digestion and ligation method, following the same procedure as described for the construction of pDR196-*A07.SUC2.* Positive clones were identified by PCR using the primers pC1300s-F/R, and the resulting recombinant plasmids were designated pC1300s-*SUC2* and pC1300s*-MYC2*, respectively. Primer sequences are listed in S3 Data. These overexpression vectors were used to transform oilseed rape and *A. thaliana*.

### Hairy root induction in oilseed rape

Surface-sterilized oilseed rape seeds were sown on MS solid medium, and sterile seedlings were grown in a light incubator. Leaves from one-week-old seedlings with the first pair of true leaves fully expanded were excised, cut into 1 cm × 1 cm explants, and pre-cultured on MS medium in the dark for 2 days.

The pC1300s-*SUC2* plasmid was introduced into *Agrobacterium rhizogenes* strain Ar1193 (Vazyme, Nanjing, China) using the same method as for GV3101 transformation. Positive Ar1193 strains were activated in LB liquid medium to an OD₆₀₀ of 0.6, collected and resuspended in sterile MS liquid medium. The pre-cultured explants were immersed in the bacterial suspension for 10 min with gentle agitation, blotted dry, and then co-cultivated on MS medium containing 200 mg/L acetosyringone at 25°C in the dark for 2 days.

After co-cultivation, the explants were washed with MS liquid medium containing 500 mg/L cefotaxime and transferred to MS solid medium with the same concentration of cefotaxime for hairy root induction under light conditions. Two weeks later, emerging hairy roots were excised and cultured individually on fresh MS medium with cefotaxime. Genomic DNA was extracted using the HiPure Plant Genomic DNA Kit (Magen, Guangzhou, China), and PCR (primers: *rolA*-F/R) amplification of the *rolA* gene was performed to confirm successful transformation. Positive transgenic lines were identified by PCR (primers: pC1300S-F/R) and qPCR analysis. Primer sequences are listed in S3 Data. Detailed information on the expression lines is provided in S4 Data.

### Genetic transformation of *A. thaliana*

The overexpression vectors pC1300s-*SUC2* and pC1300s-*MYC2* were transformed into *A. tumefaciens* GV3101 by the freeze-thaw method. Positive transformants were identified by colony PCR with primers pC1300S-F/R. A positive colony was inoculated into 2 mL LB (50 μg/mL kanamycin and 20 μg/mL rifampicin) and cultured at 28°C, 180 rpm for 24 h. One milliliter was then transferred to 100 mL LB with the same antibiotics and cultured to OD_600_ = 0.8-1.0. Cells were harvested, washed once with 1/2 MS medium, and resuspended in infiltration buffer (1/2 MS with 5% sucrose and 0.03% Silwet L-77) to OD_600_ = 0.6, then incubated on ice for 30 min.

*A. thaliana* at bolting to full-flowering stage (4–5 weeks old) were used. One day before infiltration, the substrate was watered thoroughly, and all siliques and open flowers were removed. Inflorescences were submerged in the bacterial suspension for 1 min with gentle agitation. Excess solution was drained off, and plants were covered with plastic wrap to maintain humidity, followed by dark incubation for 24 h. The cover was then removed, and plants were returned to normal light with regular watering. Infiltration was repeated once a week two to three times. Mature seeds were harvested and dried at 37°C for one week for transformant screening.

The pC1300s-*SUC2* construct was transformed into the *Atsuc2* heterozygous mutant. T_0_ seeds were sown on selection medium (1/2 MS with 3% sucrose) containing 50 mg/L hygromycin B. After 7–10 days, surviving seedlings were confirmed by PCR (primers pC1300S-F/R) to carry the *AtSUC2/Atsuc2/BnaA07.SUC2* genotype. Positive plants were bagged and self-pollinated to produce T_1_ seeds, which were similarly selected for the same genotype and used for clubroot resistance assays. Detailed information on the expression lines is provided in S4 Data.

The pC1300s-*MYC2* construct was transformed into the *Atmyc2* homozygous mutant. Positive transformants carrying the *Atmyc2/BnaA05.MYC2* genotype were selected on hygromycin-containing medium and confirmed by PCR (primers pC1300S-F/R). These plants were bagged and self-pollinated to obtain T_1_ seeds, which were then selected for the same genotype and used for clubroot resistance assays. Detailed information on the expression lines is provided in S4 Data.

### CRISPR/Cas9 vector construction

A specific target site was designed using the CRISPR-P v2.0 tool (http://crispr.hzau.edu.cn/cgi-bin/CRISPR2/) at position 491 of the first exon of *BnaA07.SUC2*, with the 20 bp target sequence TCTTGGCCGATCTATCCGCG (excluding the PAM GGG). Oligonucleotide primers sgRNA-*SUC2*-F and sgRNA-*SUC2*-R (S3 Data) were designed and synthesized, each containing overhangs complementary to *BsaI*-digested ends for sgRNA expression cassette construction.

The pSKE401 vector was linearized by *Bsa*I (EC203; TransGen Biotech) digestion, gel-purified, and the oligonucleotides were annealed (95°C for 5 min, then cooled to 25°C at 0.1°C/s). The annealed product was ligated into the linearized vector at a molar ratio of 1:3 (vector:insert) using T4 DNA ligase (FL101–01; TransGen Biotech) at 16°C for 1 h. The ligation product was transformed into DH5α competent cells, and transformants were selected on LB agar containing kanamycin (50 μg/mL). Positive clones were identified by colony PCR with primer U6-F and verified by sequencing (Sangon Biotech, Shanghai, China). The verified recombinant plasmid was designated pSKE401-*SUC2*, in which the sgRNA is driven by the U6 promoter and Cas9 by the 35S promoter. The knockout vector was ultimately used to transform oilseed rape.

### Genetic transformation of oilseed rape

The gene knockout vector pSKE401-*SUC2* was transformed into *A. tumefaciens* strain GV3101, and positive transformants were identified using the primer pair pSKE401-F/R (S3 Data). *A. tumefaciens* GV3101 strains harboring either the overexpression or gene knockout vectors were inoculated into LB liquid medium and cultured with shaking until the OD_600_ = 0.6. Bacterial cells were harvested by centrifugation and resuspended in sterile MS liquid medium for subsequent use.

Seeds of oilseed rape (Westar) were surface-sterilized and germinated on MS medium. Under sterile conditions, leaves and hypocotyls from one-week-old seedlings were cut into explants and immersed in *A. tumefaciens* (positive strains containing pSKE401-*SUC2* or pC1300s-*SUC2* or pC1300s-*MYC2*) suspension for 10 min with gentle agitation. Explants were then transferred to M1 medium for co-cultivation at 24°C in the dark for 48 h. After co-cultivation, explants were transferred to M2 selection medium and cultured at 24°C under a 16 h light/8 h dark photoperiod for 15–20 days. The same conditions were used for subsequent differentiation and rooting stages. Explants were then transferred to M3 differentiation medium to induce shoot formation, with subculturing every 20 days until shoots emerged. Once shoot apical meristems formed, shoots were excised from callus under sterile conditions and transferred to M4 rooting medium. Rooted plantlets were placed in a flask with water, covered with plastic film for acclimatization in the greenhouse for three days, and then transferred to soil for normal growth.

For T_0_ transgenic oilseed rape plants harboring the overexpression construct, PCR (pC1300S-F/R) and qPCR analyses were performed to identify positive individuals. These plants were bagged individually for self-pollination to obtain T_1_ seeds, which were then reconfirmed for the transgene. Overexpression lines were selected for subsequent studies. Detailed information on the expression lines is provided in S4 Data.

For T_0_ transgenic oilseed rape plants carrying the gene-editing construct, the target site was amplified by PCR using primers KO-*SUC2*-F/R to detect editing events. Positive plants with confirmed edits were bagged and self-pollinated to obtain T_1_ progeny. T_1_ plants were then screened by PCR with primers KO-*SUC2*-F/R and Cas9-F/R to identify lines that were homozygous for the edited allele and lacked the Cas9 cassette. These lines were further self-pollinated to generate T_2_ progeny for subsequent experimental validation. Detailed information on the expression lines is provided in S4 Data.

### qPCR analysis

For RT-qPCR, first-strand cDNA was synthesized from total RNA using the Hifair III 1st Strand cDNA Synthesis Kit (gDNA digester plus; YEASEN) according to the manufacturer’s protocol. Quantitative PCR was performed using BlasTaq 2X qPCR Master Mix (ABM, Canada) on Bio-Rad CFX96 Real-Time System (Bio-Rad, USA).

The relative expression level of genes was calculated using the 2^-ΔΔCT^ method. The primers are listed in S3 Data. Three technical replicates were performed for each biological sample [60].

### Sugar extraction and quantification

Root samples from non-inoculated *BnaA07.SUC2*-OE#1 lines and wild-type controls at 40 days after germination were flash-frozen in liquid nitrogen and ground into a fine powder. Metabolites were extracted with 1 mL of 70% (v/v) acetonitrile by ultrasonication (40 kHz, 10 min). After centrifugation (12,000 × g, 10 min, 4°C), the supernatants were passed through a 0.22 μm membrane filter. Sugar quantification was performed using LC-MS/MS on a SCIEX Triple Quad 5500 system coupled to an ultra-performance liquid chromatography (UPLC) unit and an online photodiode array detector (Shimadzu).

### Fourier-transform infrared (FTIR) microspectroscopy analysis

For the oilseed rape overexpression line OE1 (*BnaA07.SUC2*#OE1) and wild-type (WT), roots were embedded in OCT compound (Biodium) and sectioned into 5 μm slices. FTIR microspectroscopy was performed on three biological replicates per genotype using a Nicolet iN10 microscope (Thermo Scientific) with the following settings: spectral range of 4000–674 cm ⁻ ¹, spatial resolution of 24 × 24 μm, spectral resolution of 8 cm ⁻ ¹, and 128 scans per spectrum. Spectral analysis focused on the carbohydrate region (1150–1000 cm ⁻ ¹), where peak areas were quantified using OMNIC Picta software.

### Measurement of clubroot diameter

The maximum diameter of the inoculated *A. thaliana* and oilseed rape primary roots was measured as the clubroot diameter.

### *P. brassicae* biomass quantification

Total genomic DNA was extracted from whole infected roots using the HiPure Plant Genomic DNA Kit (Magen, Guangzhou, China). Quantitative real-time PCR (qPCR) was performed using SYBR Green Master Mix on a Bio-Rad CFX96 Touch Real-Time PCR System. The thermal cycling conditions were: 95°C for 30 s, followed by 40 cycles of 95°C for 5 s and 60°C for 30 s, with a melt curve stage from 65°C to 95°C (increment 0.5°C per 5 s). The relative biomass of *P. brassicae* was represented by the content of the *P. brassicae ACTIN* gene (AY452179.1) relative to that of the *A. thaliana ACTIN* gene (AT3G18780) or *B. napus ACTIN* gene (BnaC02g00690D). The primers are listed in S3 Data. Relative biomass of *P. brassicae* was calculated using the 2^–ΔΔCt^ method.

### Disease assessment

For disease evaluation, clubroot symptoms were assessed using a 0–5 rating scale: 0, no visible galls; 1, small galls mainly on lateral roots; 3, distinct galls on primary roots with inhibited plant growth; and 5, severe galls covering the entire root system and extending to the hypocotyl, accompanied by severe growth inhibition. The disease index was calculated from three biological replicates, each comprising at least 30 plants [61].


$$\text{DI }(\%)\;=\;\frac{\sum (\text{rating class})\times (\#\text{plants in rating class})}{(\text{total}\#\text{plants in treatment})\times \text{5}}\;\times \;\text{1}\text{0}\text{0}$$

### Yeast one-hybrid (Y1H) assay

The Y1H cDNA library (OE Biotech, Shanghai) was constructed using mRNA isolated from *P. brassicae*-infected oilseed rape clubroot tissue. A 1.2-kb promoter fragment of *BnaA07.SUC2* was amplified using the primer pair pABAi-*SUC2*-F/R, digested with *Sac*I and *Xho*I, and cloned into the pAbAi vector to generate the bait construct. Positive clones were identified using the primers pAbAi-F/R. Primer sequences are listed in S3 Data.

The recombinant vector was then transformed into the *S. cerevisiae* Y1HGold strain. Positive transformants were selected on SD/-Leu plates and validated by AbA resistance screening. The yeast cDNA library was screened against the bait by mating assay (Y1HGold × Y187). Positive yeast clones from the mating assay were resuspended, and the prey plasmids were rescued via transformation into *E. coli* DH5α. Each rescued prey plasmid was then co-transformed with the pAbAi-bait construct into fresh Y1HGold cells. Finally, protein-DNA interactions were confirmed by assessing yeast growth on SD/-Leu plates supplemented with 300 ng/mL AbA.

### Dual-luciferase reporter assay (Dual-LUC)

The 1.2-kb promoter fragment upstream of *BnaA07.SUC2* ATG was amplified with primers 0800-SUC2-F/R and gel-purified. The 0800-LUC vector was linearized by *Kpn*I and *Bam*HI double digestion, then mixed with the purified fragment at a 2:1 molar ratio (insert:vector) in a homologous recombination reaction at 50°C for 15 min. The reaction was transformed into *E. coli* DH5α competent cells, plated on kanamycin agar, and incubated overnight at 37°C. Positive clones were identified by colony PCR with primers 0800LUC-F/R, yielding the recombinant plasmid 0800LUC-*SUC2*. The same method was used to construct 62SK-*MYC2*. Primer sequences are listed in S3 Data.

The recombinant plasmids were transformed into *A. tumefaciens* strain GV3101, and positive clones were selected. The infiltration procedure in *N. benthamiana* leaves was performed as described for subcellular localization assays. For imaging, leaves were sprayed with 1 mM D‑luciferin, and luminescence signals were captured using a CCD camera (IVIS system) after dark adaptation. For activity assays, infiltrated leaf discs were harvested, ground in liquid nitrogen, and luciferase activity was measured using the Dual‑Luciferase Reporter Assay System (Promega) on a GloMax 20/20 Luminometer (Promega). Relative luciferase activity was calculated as the ratio of firefly luciferase (LUC) to Renilla luciferase (REN).

### Electrophoretic mobility shift assay (EMSA)

The coding sequence of *BnaA05.MYC2* was amplified using the primer pair His-MYC2-F/R, digested with *Eco*RI and *Eag*I, and cloned into the pET-28a(+) vector. Positive clones were identified using the primers pET-28a-F/R. Primer sequences are listed in S3 Data.

The recombinant vector was transformed into *E. coli* strain BL21 for protein expression. The expression of His-tagged BnaA05.MYC2 fusion protein was induced, and the protein was purified using a nickel‑nitrilotriacetic acid (Ni‑NTA) resin affinity chromatography kit (MagneHis Protein Purification System, Promega) according to the manufacturer’s instructions. For EMSA, biotin-labeled double-stranded probes containing the predicted MYC2 binding site from the *BnaA07.SUC2* promoter (S3 Data) were synthesized. Binding reactions containing the labeled probe and purified protein were incubated in EMSA buffer for 20 min at room temperature. For competition assays, 50- or 200-fold of unlabeled competitor or mutated probe was included. Protein-DNA complexes were resolved on a 6% native polyacrylamide gel in 0.5 × Tris-Borate-EDTA buffer (TBE) at 100 V for approximately 1 h and visualized using a chemiluminescent detection system (Typhoon scanner).

### Methyl jasmonate (MeJA) treatments

MeJA powder was dissolved in dimethyl sulfoxide (DMSO) to prepare a 1 M stock solution. The stock solution was then diluted with distilled water to a final working concentration of 100 μM MeJA. Oilseed rape plants were grown in soil in a greenhouse until the five-leaf stage. For MeJA treatment, the 100 μM MeJA working solution was applied to the root zone of each seedling as a drench. Control plants received an equal volume of mock solution. Whole root tissues were harvested from both MeJA-treated and mock-treated plants at 1, 3, 6, 12, and 24 h post-treatment for subsequent analysis of *BnaA05.MYC2* expression.

### JA and JA-Ile content detection

Whole root tissues were flash-frozen in liquid nitrogen and ground to a fine powder. Metabolites were extracted in 1 mL of ice-cold 75% methanol by ultrasonication (40 kHz, 10 min). After centrifugation (5,000 × g, 10 min, 4°C), the supernatant was collected. The pellet was re-extracted with 0.5 mL of 75% methanol, and the combined supernatants were passed through a 0.22 μm filter. JA and JA-Ile were quantified by LC-MS/MS using a SCIEX Triple Quad 5500 system coupled with a UPLC unit and an inline photodiode array detector.

### Statistical analysis

Data analysis was performed using SPSS (v26.0, IBM) for orthogonal experimental design analysis, GraphPad Prism (v8.0) for general statistical tests, and Microsoft Excel 365 for data organization. Prior to parametric analysis, the assumptions of normality and homogeneity of variances were assessed. When these assumptions were met, specific comparisons were assessed as follows: unpaired, two-tailed Student’s t-tests for individual treatment versus control comparisons; one-way ANOVA with Tukey’s post-hoc test for all pairwise comparisons; one-way ANOVA with Dunnett’s post-hoc test or Dunnett’s T3 for comparisons of multiple treatments against a single control; and two-way ANOVA followed by Tukey’s multiple comparisons test for experiments involving multiple interdependent variables. Significance levels are denoted as *P < 0.05, **P < 0.01, and ***P < 0.001.

## Supporting information

S1 Fig Histocytological observation of the clubroot in oilseed rape. (DOCX)

S2 Fig Transcripts per million (TPM) values of six candidate *SUC* genes at 21 and 28 days post-inoculation (dpi). (DOCX)

S3 Fig Transport activity of BnaA07. SUC2 in*Xenopus laevis* oocytes. (DOCX)

S4 Fig Functional validation of *BnaA07.SUC2* in promoting susceptibility in transgenic oilseed rape hairy roots. (DOCX)

S5 Fig Expression of *BnaA07.SUC2* in *A. thaliana* complementation lines and corresponding disease index. (DOCX)

S6 Fig Overexpression of *BnaA07.SUC2* in oilseed rape promotes sucrose content in roots. (DOCX)

S7 Fig Functional validation of *BnaA05.MYC2* in clubroot resistance in *A. thaliana.* (DOCX)

S1 Data Transcripts per million (TPM) values of oilseed rape *SUC* genes derived from transcriptome sequencing of *P. brassicae*-infected oilseed rape roots. (XLSX)

S2 Data Transcripts per million (TPM) values of oilseed rape JA pathway genes derived from transcriptome sequencing of *P. brassicae*-infected oilseed rape roots. (XLSX)

S3 Data All primers used in this study. (XLSX)

S4 Data Summary of transformed lines used in this study. (XLSX)
