# Supplementary material for: BnaA07.SUC2 regulated by BnaA05.MYC2 in jasmonate pathway promotes oilseed rape susceptibility to Plasmodiophora brassicae
Source: PLoS Pathog. 2026 May 5;22(5):e1014199. doi: 10.1371/journal.ppat.1014199 (PMC13143063; doi:10.1371/journal.ppat.1014199)
Supplement: S1 Fig — (DOCX) [file ppat.1014199.s001.docx]

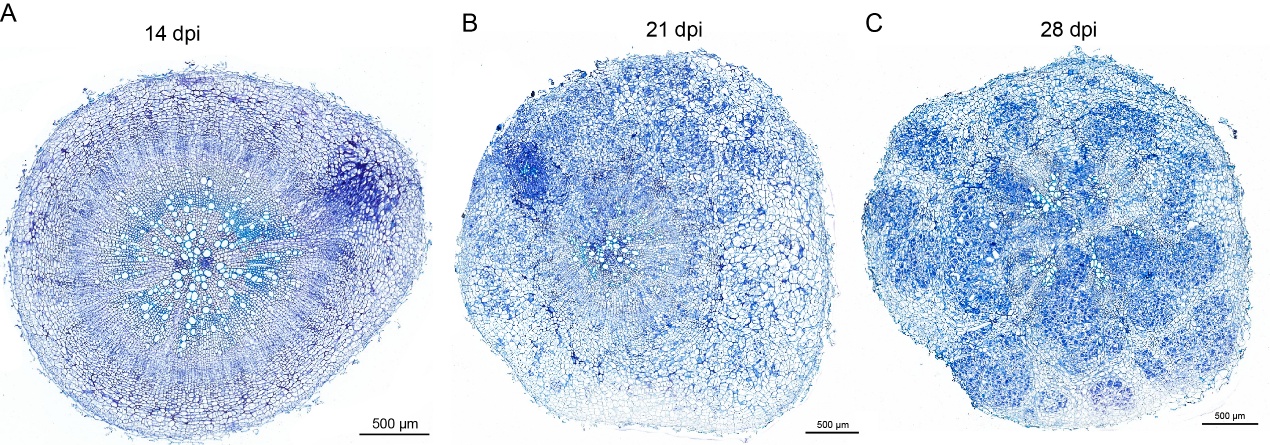


**S1 Fig. Histocytological observation of the clubroot in oilseed rape.**

Toluidine blue-stained paraffin cross sections of clubroot in oilseed rape at 14 days post-inoculation (dpi) (A), 21 dpi (B), 28 dpi (C) Scale bar = 500 μm.
