# Supplementary material for: BnaA07.SUC2 regulated by BnaA05.MYC2 in jasmonate pathway promotes oilseed rape susceptibility to Plasmodiophora brassicae
Source: PLoS Pathog. 2026 May 5;22(5):e1014199. doi: 10.1371/journal.ppat.1014199 (PMC13143063; doi:10.1371/journal.ppat.1014199)
Supplement: S2 Fig — (DOCX) [file ppat.1014199.s002.docx]

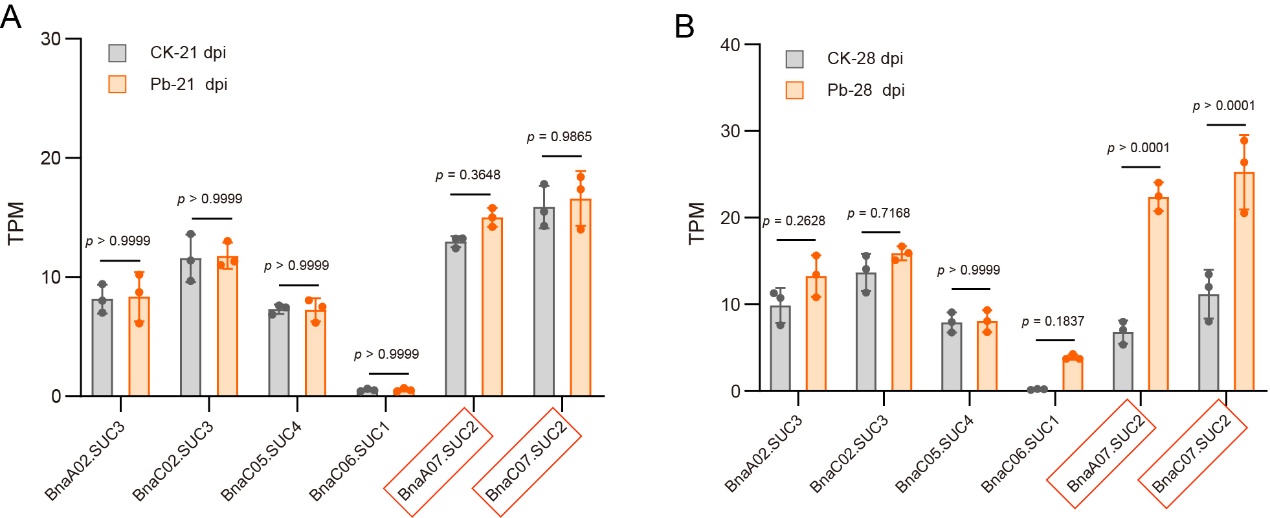


**S2 Fig. Transcripts per million (TPM) values of six candidate *SUC* genes at 21 and 28 days post-inoculation (dpi).**

TPM values for 21 dpi (A) and 28 dpi (B) are shown. Data are obtained from transcriptome sequencing of *P. brassicae*-infected oilseed rape roots. Data are presented as means ± SD (n = 3). **P* < 0.05 (two-way ANOVA, Tukey’s test). Genes in red boxes are significantly upregulated at 21 dpi and 28 dpi compared to the corresponding time-point controls.
