# Supplementary material for: BnaA07.SUC2 regulated by BnaA05.MYC2 in jasmonate pathway promotes oilseed rape susceptibility to Plasmodiophora brassicae
Source: PLoS Pathog. 2026 May 5;22(5):e1014199. doi: 10.1371/journal.ppat.1014199 (PMC13143063; doi:10.1371/journal.ppat.1014199)
Supplement: S3 Fig — (DOCX) [file ppat.1014199.s003.docx]

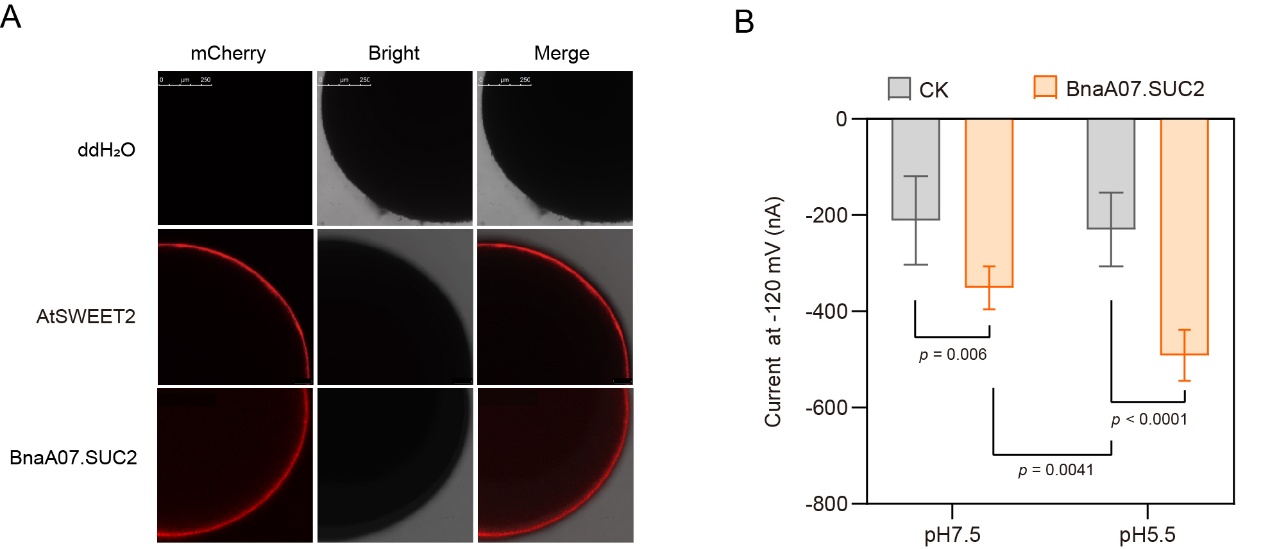


**S3 Fig. Transport activity of BnaA07.SUC2 in *Xenopus laevis* oocytes.**

(A) Subcellular localization of mCherry-tagged BnaA07.SUC2 in oocytes following microinjection of *in vitro*-transcribed cRNA, visualized by confocal microscopy. Water-injected oocytes served as controls. Scale bar = 75 μm. (B) Voltage-clamp recordings of sucrose-elicited currents at -120 mV from oocytes expressing *BnaA07.SUC2* and water-injected controls (CK) under pH 5.5 and pH 7.5 conditions. Data are presented as means ± SE (n = 6). **P* < 0.05 (two-way ANOVA with Tukey’s test).
