# Supplementary material for: BnaA07.SUC2 regulated by BnaA05.MYC2 in jasmonate pathway promotes oilseed rape susceptibility to Plasmodiophora brassicae
Source: PLoS Pathog. 2026 May 5;22(5):e1014199. doi: 10.1371/journal.ppat.1014199 (PMC13143063; doi:10.1371/journal.ppat.1014199)
Supplement: S4 Fig — (DOCX) [file ppat.1014199.s004.docx]

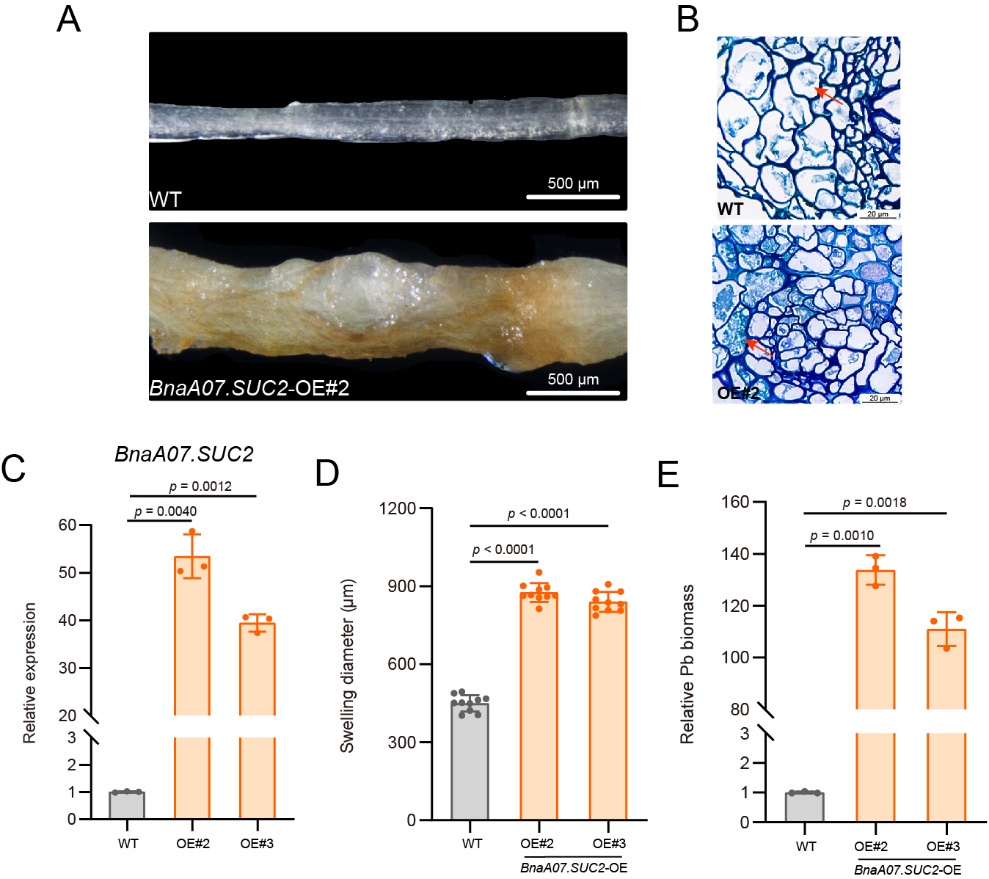


**S4 Fig. Functional validation of *BnaA07.SUC2* in promoting susceptibility in transgenic oilseed rape hairy roots.**

(A) Phenotypes of infected overexpression (OE) oilseed rape hairy root lines compared with WT at 28 dpi. Scale bars = 500 μm. (B) Toluidine blue-stained paraffin-embedded cross sections of hairy roots from samples in (A). Scale bars = 20 μm. Red arrows indicate *P. brassicae* within root cells. (C) Expression levels of *BnaA07.SUC2* in two independent OE hairy root lines relative to WT. Data represented as mean ± SD (n = 3). **P* < 0.05 (one-way ANOVA with Dunnett T3’s test). (D)–(E) Disease quantification in WT and OE hairy root lines at 28 dpi. (D) Swelling diameter and (E) relative *P. brassicae* biomass of OE hairy root lines compared with WT at 28 dpi. Data are presented as means ± SD (n = 10 for diameter; n = 3 for biomass). **P* < 0.05 (one-way ANOVA with Dunnett T3’s test).
