# Supplementary material for: BnaA07.SUC2 regulated by BnaA05.MYC2 in jasmonate pathway promotes oilseed rape susceptibility to Plasmodiophora brassicae
Source: PLoS Pathog. 2026 May 5;22(5):e1014199. doi: 10.1371/journal.ppat.1014199 (PMC13143063; doi:10.1371/journal.ppat.1014199)
Supplement: S5 Fig — (DOCX) [file ppat.1014199.s005.docx]

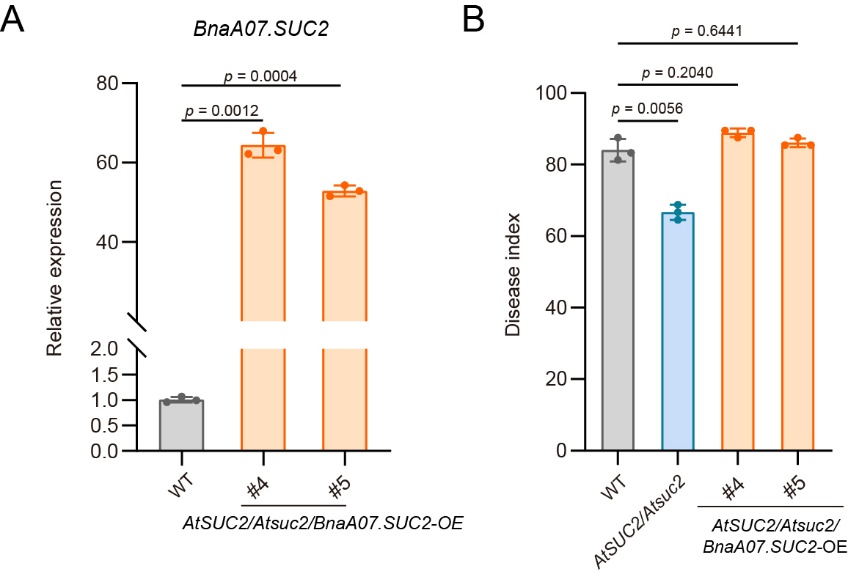


**S5 Fig. Expression of *BnaA07.SUC2* in *A. thaliana* complementation lines and corresponding disease index.**

(A) *BnaA07.SUC2* expression in *A. thaliana* complementary lines relative to WT (Col-0). Data are presented as mean ± SD (n = 3). **P* < 0.05 (one-way ANOVA with Dunnett T3’s test). (B) Disease index of *AtSUC2/Atsuc2* and *AtSUC2/Atsuc2/BnaA07.SUC2* compare with WT. Data are presented as mean ± SD (n = 3). **P* < 0.05 (one-way ANOVA with Dunnett T3’s test).
