# Supplementary material for: BnaA07.SUC2 regulated by BnaA05.MYC2 in jasmonate pathway promotes oilseed rape susceptibility to Plasmodiophora brassicae
Source: PLoS Pathog. 2026 May 5;22(5):e1014199. doi: 10.1371/journal.ppat.1014199 (PMC13143063; doi:10.1371/journal.ppat.1014199)
Supplement: S6 Fig — (DOCX) [file ppat.1014199.s006.docx]

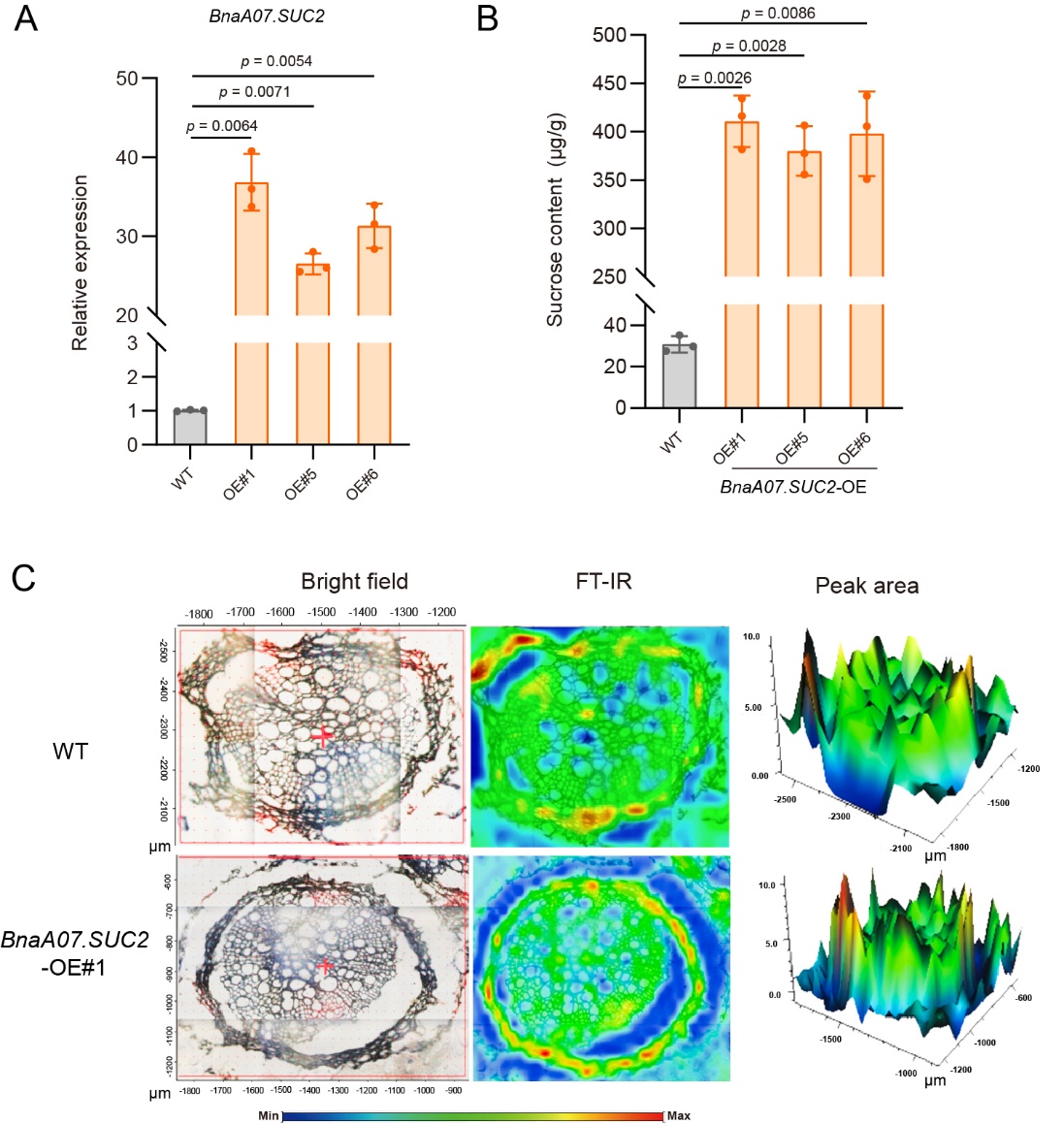


**S6 Fig. Overexpression of *BnaA07.SUC2* in oilseed rape promotes sucrose content in roots.**

(A) Expression levels of *BnaA07.SUC2* in OE lines relative to WT. Data represented as mean ± SD (n = 3). **P* < 0.05 (one-way ANOVA, Dunnett T3’s test). (B) Sucrose content in non-inoculated roots of *BnaA07.SUC2*-OE#1 lines compared with WT plants. Data represent mean ± SD (n = 3). **P* < 0.05 (one-way ANOVA, Dunnett T3’s test). (C) FTIR spectral imaging of cryo-sectioned roots from *BnaA07.SUC2*-OE#1 compared with WT. Yellow/red indicating carbohydrate accumulation.
