# Supplementary material for: BnaA07.SUC2 regulated by BnaA05.MYC2 in jasmonate pathway promotes oilseed rape susceptibility to Plasmodiophora brassicae
Source: PLoS Pathog. 2026 May 5;22(5):e1014199. doi: 10.1371/journal.ppat.1014199 (PMC13143063; doi:10.1371/journal.ppat.1014199)
Supplement: S7 Fig — (DOCX) [file ppat.1014199.s007.docx]

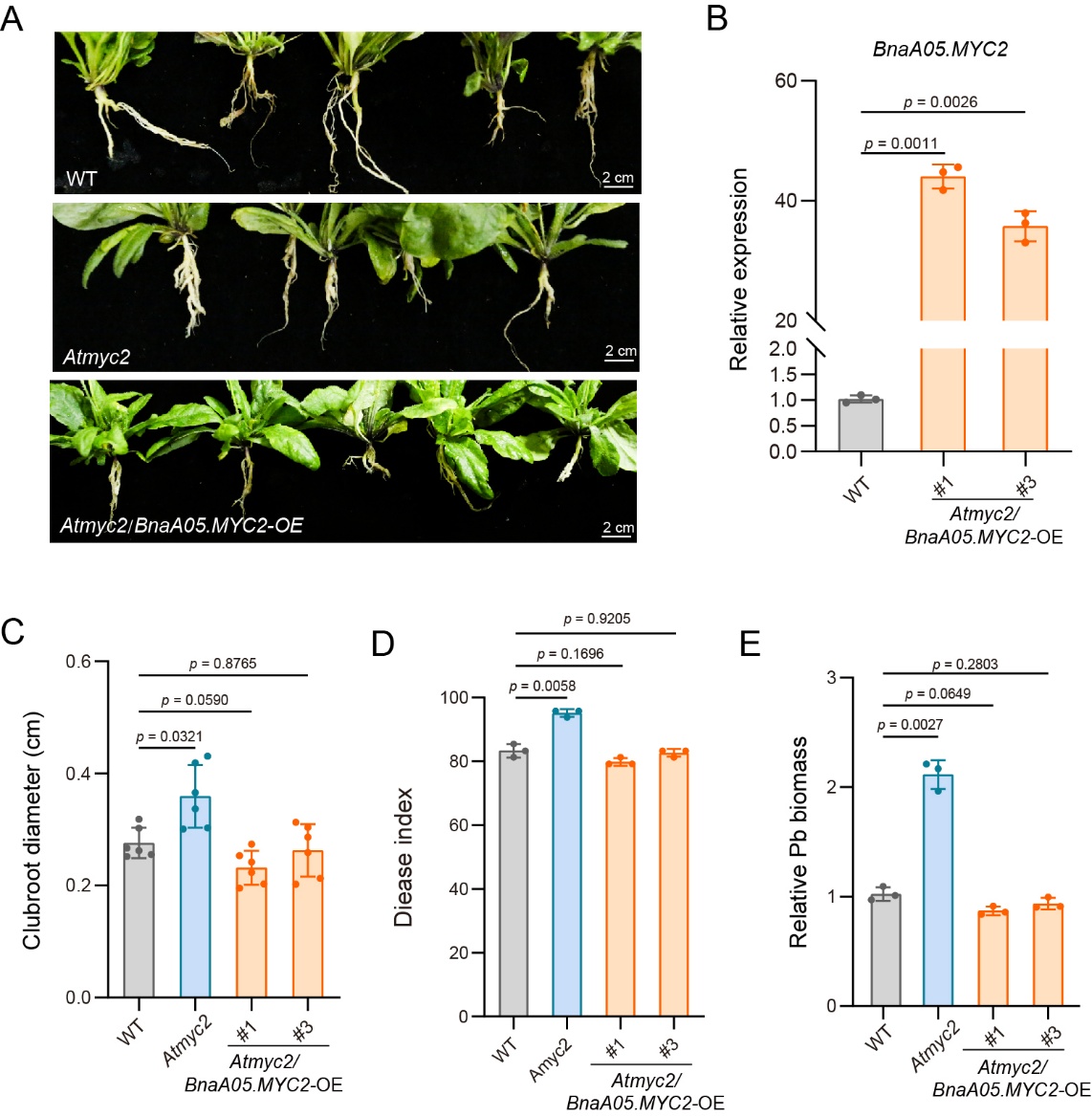


**S7 Fig. Functional validation of *BnaA05.MYC2* in clubroot resistance in *A. thaliana*.**

(A) Phenotypes of *A. thaliana* at 28 dpi: wild-type (Col-0), *Atmyc2* mutant, and *Atmyc2* mutant overexpressing *BnaA05.MYC2* (*Atmyc2*/*BnaA05.MYC2-*OE). Scale bar = 2 cm. (B) *BnaA05.MYC2* expression in *A. thaliana* complementary lines relative to WT (Col-0). Data are presented as mean ± SD (n = 3). **P* < 0.05 (one-way ANOVA with Dunnett T3’s test). (C)-(E) Disease quantification of *Atmyc2* and *Atmyc2*/*BnaA05.MYC2-*OE lines compared with WT at 28 dpi. (C) Clubroot diameters, (D) disease index (E) relative *P. brassicae* biomass. Data are presented as mean ± SD (n = 6 for diameter; n = 3 for disease index and biomass). **P* < 0.05 (one-way ANOVA with Dunnett T3’s test).
